# Erratum to: Transcriptional mapping of the primary somatosensory cortex upon sensory deprivation

**DOI:** 10.1093/gigascience/giy040

**Published:** 2018-10-27

**Authors:** Koen Kole, Yutaro Komuro, Jan Provaznik, Jelena Pistolic, Vladimir Benes, Tiesinga Paul, Tansu Celikel

In the final publication of “Transcriptional mapping of the primary somatosensory cortex upon sensory deprivation” by Koen Kole et al.,[1] the publication month for reference 15 was incorrect. The publication month for reference 15 has been corrected and the corrected manuscript is published in *GigaScience*, Volume 6, Issue 10.
