# Supplementary material for: Transcriptional mapping of the primary somatosensory cortex upon sensory deprivation
Source: Gigascience. 2017 Aug 23;6(10):1–6. doi: 10.1093/gigascience/gix081 (PMC5965344; doi:10.1093/gigascience/gix081)
Supplement: GIGA-D-16-00170_Revision-2.pdf [file gix081_GIGA-D-16-00170_Revision-2.pdf]

Revised manuscript Click here to download Manuscript Gigascience_Kole_RevisedRound2.pdf

Transcriptional mapping of the primary somatosensory cortex upon sensory deprivation

Koen Kole[1,2], Yutaro Komuro[1], Jan Provaznik[3], Jelena Pistolic[3], Vladimir Benes[3], Paul Tiesinga[2], Tansu Celikel[1]

(1) Department of Neurophysiology, (2) Department of Neuroinformatics, Donders Institute for Brain, Cognition, and Behaviour, Radboud University, Nijmegen - the Netherlands. (3) European Molecular Biology Laboratory (EMBL), Genomics Core Facility, Heidelberg - Germany

E-mail addresses (in the order of appearance): k.kole@neurophysiology.nl, y.komuro@neurophysiology.nl, jan.provaznik@embl.de, jelena.pistolic@embl.de, benes@embl.de, p.tiesinga@science.ru.nl, celikel@neurophysiology.nl (corresponding author)

Background (138)

Experience-dependent plasticity (EDP) is essential for anatomical and functional maturation of sensory circuits during development. Although the principal synaptic and circuit mechanisms of EDP are increasingly well studied experimentally and computationally, its molecular mechanisms remain largely elusive. EDP can be readily studied in the rodent barrel cortex, where each ‘barrel column’ preferentially represents deflections of its own principal whisker. Depriving select whiskers while sparing their neighbours introduces competition between barrel columns, ultimately leading to weakening of intracortical, translaminar (i.e. Cortical Layer (L)4-to-L2/3) feed-forward excitatory projections in the deprived columns. The same synapses are potentiated in the neighbouring spared columns. These experience-dependent alterations of synaptic strength are thought to underlie somatosensory map plasticity. We used RNA sequencing in this model system to uncover cortical-column and -layer specific changes on the transcriptome level that are induced by altered sensory experience.

Findings (66)

Column- and layer-specific barrel cortical tissues were collected from juvenile mice with all whiskers intact and mice that received 11-12 days long whisker (C-row) deprivation before high quality RNA was purified and sequenced. The current dataset entails an average of 50 million paired-end reads per sample, 75 base pairs in length. On average, 90.15% of reads could be uniquely mapped to the mm10 reference mouse genome.

Conclusions (32) – Word total for the abstract: 246 out of 250

The current data reveal the transcriptional changes in gene expression in the barrel cortex upon altered sensory experience in juvenile mice and will help to molecularly map the mechanisms of cortical plasticity.

Data Description

Context

Sensory experience powerfully shapes neural circuits. Changes due to sensory organ deprivation such as eye closure, digit amputation, and whisker trimming provide powerful means for studying mechanisms of experience dependent cortical plasticity.

In the whisker system experience dependent plasticity is most commonly studied in the barrel cortex subfield of the primary somatosensory cortex where neural representations of whiskers change in response to altered patterns of incoming sensory information. As originally shown in the barrel cortex [1] sensory deprivation induced by transient whisker trimming is sufficient to perturb neural receptive fields both during development and in adulthood. Previous work has also shown that the cellular basis of deprivation-induced decreases in whisker evoked representations are primarily due to a reduction of synaptic strength in monosynaptically connected feed-forward neuronal networks in behaving animals [2, 3]. Conversely whisker sparing induced enhancement in whisker representation is mediated at least in part by the long-term synaptic facilitation expressed along the L4 projections *in vivo* [4]. Identification of the molecular events that mediate these bidirectional changes in synaptic connectivity will benefit from systematic analysis of the gene transcription. Therefore, we performed RNA sequencing in the barrel cortex with or without sensory deprivation across cortical layers 2-4. This database will assist molecular and cellular neurobiologists in addressing the molecular mechanisms associated with experience dependent plasticity, and will enable statistical approaches to determine the dynamics of the coupled changes across molecular pathways as cortical circuits undergo plastic changes in their organization.

Methods

Animals

All experiments were performed in accordance with the Animal Ethics Committee of the Radboud University in Nijmegen, the Netherlands. Pregnant wild type mice (Charles River, stock number 000664) [RRID:NCBITaxon_10090] were kept at a 12-hour light/dark cycle with access to food *ad libitum*. Cages were checked for birth daily. To induce experience-dependent plasticity, pups underwent bilateral plucking of their C-row whiskers under isoflurane anaesthesia at P12 (**Figure 1**). Control animals were not plucked but anaesthetized and handled similarly. After recovery pups were returned to their home cage. Every other day pups were checked for whisker regrowth, which were plucked if present. At P23-P24, pups were randomly selected from their litter for slice preparation and tissue collection. For each experimental condition (i.e. whisker deprived or control), 4 female pups were used, thus each group consisted of 4 independent biological samples (also known as biological replicates). Samples from cortical layer (L) 4 and L2/3 were treated independently with their own corresponding groups of control, deprived, 1st order spared, 2nd order spared columns as detailed in Figure 1.

Figure 1 is about here

Slice preparation and sample collection

Pups were anaesthetized using isoflurane and then perfused with ice-cold carbogenated slicing medium (108 mM ChCl, 3 mM KCl, 26 mM $NaHCO_3$, 1.25 mM NaH_2PO_4, 25 mM glucose, 1 mM $CalCl_2$, 6 mM $MgSO_4$ and 3 mM Na-pyruvate). Next, pups were decapitated before the brain was quickly dissected out and 400 µm thalamocortical slices from each hemisphere were prepared as described before [2, 3]. Slices were transferred to 37 degrees Celsius carbogenated ACSF (120 mM NaCl, 3.5 mM KCl, 10 mM glucose, 2.5 mM $CaCl_2$, 1.3 mM $MgSO_4$, 25 mM $NaHCO_3$ and 1.25

mM NaH_2PO_4) where they were kept for 30 minutes and recovered at room temperature for another 30 minutes until tissue collection.

After incubation, slices were placed under a Nikon Eclipse FN1 microscope. The holding chamber was continuously perfused with room temperature carbogenated ACSF. Due to the 55 degree cut, slices were obtained in which S1 barrels from specific rows (A-E) could be identified [2]. A thin, long glass pipette was pulled using a Sutter instruments P-2000 pipette puller and was used to make intercolumnar incisions from L1 to the bottom of L4 after which the slice was placed under a binocular dissection microscope where the location of specific barrel columns could now be readily identified by eye. A sterile 32G needle was then used to cut out L2/3 and L4 separately from each column. Tissue from columns A/E and B/D were pooled as they both constitute second and first order spared whiskers, respectively. Immediately after dissection, tissue samples were snap frozen in liquid nitrogen and stored at -80 degrees Celsius until further use. All tools that came into direct contact with brain tissue were treated using RNAseZap in order to minimize RNAse contamination.

RNA isolation and quality control

Tissue samples originating from the same rows and layers were pooled within each animal. From control animals, only the C column tissues were used (also see *Re-use potential*). Tissue was quickly dissolved in Qiazol (Qiagen #79306), after which RNA was isolated using the miRNeasy Mini kit (Qiagen #217004), DNAse treated (Thermo Scientific, #EN0521) and cleaned up using RNeasy MinElute kit (Qiagen #74204), all following the manufacturer's instructions. Samples were then stored at -80 degrees Celsius until further processing.

RNA sample integrity was determined using Agilent Tapestation (High Sensitivity RNA Screentape). Sample RINs ranged from 7.1 to 8.8. To further assess RNA purity and integrity,

RNA samples were used in RT-PCR to confirm that cDNA could be produced and that a large (~1000 bp) amplicon could be obtained. To produce cDNA, SuperScript® II Reverse Transcriptase (Thermo Scientific #18064014) and random hexamer primers (Roche #11034731001) were used. The resulting cDNA was then added to a PCR reaction mix which further consisted of Jumpstart Ready Mix (Sigma P2893) and exon-exon junction spanning CamKII primers (FW TCCAACATTGTACGCCTCCAT; RV TGTTGGTGCTGTCGGAAGAT). From all cDNA samples a fragment of the expected size could be amplified, suggesting that the RNA samples contained pure RNA of sufficient integrity. All RNA samples thus passed our quality control criteria and were subjected to RNA sequencing.

RNA sequencing

RNA sequencing was conducted at the Genomics Core Facility of the EMBL, Heidelberg, Germany [RRID:SCR_004473]. The cDNA library was generated using the non-stranded NEBNext Ultra RNA Library Preparation Kit for Illumina (New England Biolabs, catalogue #E7530), which includes oligo-dT bead selection of mRNA. For library enrichment, 13-14 PCR cycles were performed. Pooled libraries were sequenced on the Illumina NextSeq 500 instrument [RRID:SCR_014983] in a 75bp paired-end mode using High output flow cells.

Data validation and quality control

Sequencing read quality was assessed using FastQC (Babraham Bioinformatics) [RRID:SCR_014583], the results of which were merged using MultiQC (http://multiqc.info) [RRID:SCR_014982]. Results are displayed in **Figure 2**. Per base quality *phred* scores range from 34.80 to 35.15, indicating base call accuracies of >99.9% (**Figure 2A**). Overall 91.48-94.03% of reads had a mean *phred* score of 30 or above (**Figure 2B**). In line with these scores, per base N content (i.e. percentage of bases that could not confidently called) was very low, with a maximum value 0.053%.

Figure 2 is about here

Reads were then mapped to the mm10 reference genome using STAR [5] [RRID:SCR_005622], which uniquely mapped between 39,000,000 and 59,000,000 reads, constituting an average 90.15% unique map rate across samples (**Figure 2D**). Since the library preparation protocol entails a PCR enrichment step, which can lead to technical duplication and hence an overestimation of observed transcripts, we used Seqmonk (Babraham Bioinformatics) [RRID:SCR_001913] to plot the read density against the duplication levels (i.e. the percentage of duplicate reads) for each transcript. The obtained duplication plots showed a clear positive relation between read density and duplication levels (**Figure 3** and **Supplemental Figure 1**), suggesting that the origin of read duplication is biological, rather than technical.

Based on the above quality control measures we determined that our RNA-sequencing data was of sufficient quality to be used in downstream analyses, therefore we continued with gene expression analysis.

Figure 3 is about here

Analysis of gene expression

Using a 2 read cut-off we identified 16,900 to 17,600 transcripts per sample (**Figure 4A**). Raw gene counts can be found online (see Supporting Data – DOI to appear). Differential gene expression analyses across groups were performed using EdgeR v3.12.1 [6, 7] [RRID:SCR_012802] using only genes with a count per million (CPM) >1 in at least 4 samples (**Supplementary Table 1** for details on the commands used). Since laminar identity is an important feature of our experimental setup, we assessed the relative expression of known

molecular markers for L2/3 (*Cacna1h*, *Id2*, *Igfbp4*, *Igfn1*, *Mdga1*, *Plcxd1*, *Rasgrf2*, *Rgs8*, *Tle3*) and L4 (*Cartpt*, *Cyp39a1*, *Kcnh5*, *Kcnip2*, *Lmo3*, *Rorb*, *Scnn1a*) [8–10], which showed selective enrichment of the laminar markers in isolated layers (**Figure 4B**).

Figure 4 is about here

To assess the variance in transcript counts, we calculated the coefficient of variation (CV) for each transcript with a cut-off of 50 as the minimal read count separately for each group (**Figure 4C**). This analysis showed that, on average, 85.93% of transcripts have a CV below 15%, suggesting low variance across transcript counts for individual genes. Principal component analysis (PCA) showed that samples cluster based on layer, and the first two components explained ~88% variance the data (**Figure 4C**, **Supplemental Figure 2B**).

These quality control routines suggest that we have obtained RNA-sequencing data of high read quality, with individual bases being called confidently throughout the length of reads, which uniquely map to the mm10 reference genome at high rates (>90% average). The laminar origin of our samples could be identified through known molecular markers, confirming our samples are of high anatomical specificity.

Re-use potential

The current RNA-seq dataset might help address the molecular underpinnings of cortical experience-dependent plasticity. For example, it could be used (1) to identify genes whose transcription is modulated in an experience-dependent manner, (2) to statistically map the transcriptional networks at laminar resolution, (3) creating synergy with the single neuron RNA-seq datasets [11, 12], to address the molecular diversity of the cortical networks, (4) combined with the proteomic analysis performed under comparable experimental conditions in the

accompanying manuscript (Kole et al, submitted), to systematically study the transcriptional and translational regulation of the genome upon altered sensory experience, and finally (5) to identify and quantify splice isoforms, given the sequencing depth of the current dataset. Since splicing and other posttranscriptional mechanisms govern which proteins are ultimately produced, combining the current transcriptomic dataset with a proteomics approach would also be of high importance.

The current dataset focuses on isolated cortical columns and layers, which are necessarily diverse samples containing neuronal and non-neuronal cell classes. In terms of experience dependent plasticity, although most previous studies focus on excitatory projections, inhibitory cells and even non-neuronal cells have been implicated in plasticity [13–15]. This heterogeneity might be particularly important for L2/3, as also shown by the principal component analysis (**Figure 4D**), given the relative diversity of cellular populations in supragranular layers and their heterogeneous connectivity patterns [16].

Researchers reusing our dataset should be aware that comparisons between control column C and spared columns (A/E, B/D) may have to be approached with caution, as this would involve two different columnar identities (whose transcriptomic dissimilarities are currently unknown), each coming from cortices that have had different sensory experience. However direct comparisons between the C columns across experimental conditions (i.e control versus deprived) as well as within-animal across-column comparisons in deprived animals control for these confounding variables.

Taken together we hope that this data will prove useful in discovering novel molecular targets responsible for cortical plasticity and will lead to targeted control of plasticity in health and disease.

References

1. Hand PJ (1892) Plasticity of the rat cortical barrel system. In: Strick P, Morrison AD (eds) Changing concepts of the nervous system. Academic Press, New York, pp 49–75
2. Allen CB, Celikel T, Feldman DE (2003) Long-term depression induced by sensory deprivation during cortical map plasticity in vivo. Nat Neurosci 6:291–9. doi: 10.1038/nn1012
3. Celikel T, Szostak VA, Feldman DE (2004) Modulation of spike timing by sensory deprivation during induction of cortical map plasticity. Nat Neurosci 7:534–41. doi: 10.1038/nn1222
4. Clem RL, Celikel T, Barth AL (2008) Ongoing in vivo experience triggers synaptic metaplasticity in the neocortex. Science 319:101–4. doi: 10.1126/science.1143808
5. Dobin A, Davis CA, Schlesinger F, Drenkow J, Zaleski C, Jha S, Batut P, Chaisson M, Gingeras TR (2013) STAR: ultrafast universal RNA-seq aligner. Bioinformatics 29:15. doi: 10.1093/bioinformatics/bts635
6. Robinson MD, McCarthy DJ, Smyth GK (2010) edgeR: a Bioconductor package for differential expression analysis of digital gene expression data. Bioinformatics 26:139. doi: 10.1093/bioinformatics/btp616
7. McCarthy DJ, Chen Y, Smyth GK (2012) Differential expression analysis of multifactor RNA-Seq experiments with respect to biological variation. Nucleic Acids Res 40:4288. doi: 10.1093/nar/gks042
8. Molyneaux BJ, Goff LA, Rinn JL, Arlotta P (2015) Genome-wide Analysis of In Vivo Transcriptional Dynamics during Pyramidal Neuron Fate Selection in Neocortex NeuroResource DeCoN : Genome-wide Analysis of In Vivo Transcriptional Dynamics during Pyramidal Neuron Fate Selection in Neocortex. 275–288.
9. Xue M, Atallah B V., Scanziani M (2014) Equalizing excitation–inhibition ratios across visual cortical neurons. Nature 511:596–600. doi: 10.1038/nature13321
10. Rowell JJ, Mallik AK, Dugas-Ford J, Ragsdale CW (2010) Molecular analysis of neocortical layer structure in the ferret. J Comp Neurol 518:3272–3289. doi: 10.1002/cne.22399
11. Zeisel A, Munoz-Manchado AB, Codeluppi S, Lonnerberg P, La Manno G, Jureus A, Marques S, Munguba H, He L, Betsholtz C, Rolny C, Castelo-Branco G, Hjerling-Leffler J, Linnarsson S (2015) Cell types in the mouse cortex and hippocampus revealed by single-cell RNA-seq. Science (80-) 347:1138–1142. doi: 10.1126/science.aaa1934
12. Tasic B, Menon V, Nguyen TN, Kim TK, Jarsky T, Yao Z, Levi B, Gray LT, Sorensen SA, Dolbeare T, Bertagnolli D, Goldy J, Shapovalova N, Parry S, Lee C, Smith K, Bernard A, Madisen L, Sunkin SM, Hawrylycz M, Koch C, Zeng H (2016) Adult mouse cortical cell taxonomy revealed by single cell transcriptomics. Nat Neurosci 19:335–346. doi: 10.1038/nn.4216
13. Tropea D, Van Wart A, Sur M (2009) Molecular mechanisms of experience-dependent plasticity in visual cortex. Philos Trans R Soc Lond B Biol Sci 364:341–55. doi: 10.1098/rstb.2008.0269
14. Kole K (2015) Experience-dependent plasticity of neurovascularization. J Neurophysiol 114:2077–9. doi: 10.1152/jn.00972.2014
15. Foeller E, Celikel T, Feldman DE (2005) Inhibitory sharpening of receptive fields contributes to whisker map plasticity in rat somatosensory cortex. J Neurophysiol 94:4387–400. doi: 10.1152/jn.00553.2005
16. Markram H, Muller E, Ramaswamy S, Reimann MW, Abdellah M, Sanchez CA, Ailamaki A, Alonso-Nanclares L, Antille N, Arsever S, Kahou GAA, Berger TK, Bilgili A, Buncic N, Chalimourda A, Chindemi G, Courcol J-D, Delalondre F, Delattre V, Druckmann S, Dumusc R, Dynes J, Eilemann S, Gal E, Gevaert ME, Ghobril J-P, Gidon A, Graham JW, Gupta A, Haenel V, Hay E, Heinis T, Hernando JB, Hines M, Kanari L, Keller D, Kenyon

J, Khazen G, Kim Y, King JG, Kisvarday Z, Kumbhar P, Lasserre S, Le Bé J-V, Magalhães BRC, Merchán-Pérez A, Meystre J, Morrice BR, Muller J, Muñoz-Céspedes A, Muralidhar S, Muthurasa K, Nachbaur D, Newton TH, Nolte M, Ovcharenko A, Palacios J, Pastor L, Perin R, Ranjan R, Riachi I, Rodríguez J-R, Riquelme JL, Rössert C, Sfyrakis K, Shi Y, Shillcock JC, Silberberg G, Silva R, Tauheed F, Telefont M, Toledo-Rodriguez M, Tränkler T, Van Geit W, Díaz JV, Walker R, Wang Y, Zaninetta SM, DeFelipe J, Hill SL, Segev I, Schürmann F (2015) Reconstruction and Simulation of Neocortical Microcircuitry. Cell 163:456–492. doi: 10.1016/j.cell.2015.09.029

Availability of the supporting data

Supporting data are available online (https://goo.gl/tBof51) and will be distributed via GigaScience DB.

Raw sequence reads were deposited in NCBI GEO.

Link: https://www.ncbi.nlm.nih.gov/geo/query/acc.cgi?acc=GSE90929

List of abbreviations

EDP	Experience dependent plasticity
L2/3	Cortical Layer 2/3, also known as supragranular layers
L4	Cortical Layer 4, i.e. granular layer

Competing interests

The authors declare no competing interests.

Author contributions

KK performed all experimental manipulations, sample acquisition, biological and bioinformatic quality controls, and prepared the tables and figures. YK and JaP performed bioinformatic analysis. JeP performed library prep. VB supervised RNA-seq. PT contributed bioinformatic analysis and co-supervised the project. TC designed and supervised the project. KK and TC wrote the manuscript. All authors edited otherwise approved the final version of the manuscript.

Figure Legends

Figure 1. Overview of the experimental design, sample collection and data organization. **(A)** Pups were bilaterally spared or deprived of off their C-row whiskers between P12 and P23-P24, when acute slices are made and column- and layer-specific tissues were excised. **(B)** RNA was isolated, checked for integrity and purity, and subsequently sequenced. **(C)** Organization of the database. Colour codes denote experimental groups. Same denominations are used in the read counts matrix file (see **Supplemental Data**).

Figure 2. FastQC and STAR output graphs for all samples. **(A-B)** *Phred* scores per base and per sequence. **(C)** Per sequence GC content. **(D)** STAR output of alignment scores.

Figure 3. Overlays of duplication plot contours, showing a positive correlation between read density and duplication levels. Depicted contours enclose 90% of the data points.

Figure 4. Gene expression analyses. **(A)** Histogram of read counts per transcript per sample. With a cut-off of 2 reads, between 16,900 and 17,600 transcripts could be identified across samples. **(B)** Relative expression of known molecular markers for cortical laminae. Layer 4 markers are enriched in samples originating from this layer; the same is true for Layer 2/3 marker expression in Layer 2/3 samples. **(C)** Cumulative plots of the coefficient of variance (CV) of individual experimental groups. Including only transcripts identified by 50 reads or more, average CVs of <15% are found in ~85% of transcripts. **(D)** Principal component analysis (PCA) showing sample clustering by layer, including only transcripts identified by at least 50 reads. Principal component (PC) 1 and 2 account for 88% of overall variance.

Supplemental Figure 1. Duplication plots for all samples, produced using SeqMonk (Babraham Bioinformatics).

Supplemental Figure 2. (A) Cumulative plots of the coefficient of variance (CV) of experimental each group, including transcripts identified by at least one read. Average CVs of <25% are found in ~85% of transcripts. **(B)** Principal component analysis (PCA) including transcripts identified by at least one read. The majority (88%) of overall variance is explained by Principal components (PC) 1 and 2.

Revised manuscript, redlined

Click here to download Manuscript Gigascience_Kole_RevisedRound2_Redlined.pdf

Transcriptional mapping of the primary somatosensory cortex upon sensory deprivation

Koen Kole[1,2], Yutaro Komuro[1], Jan Provaznik[3], Jelena Pistolic[3],

Vladimir Benes[3], Paul Tiesinga[2], Tansu Celikel[1]

(1) Department of Neurophysiology, (2) Department of Neuroinformatics, Donders Institute for Brain, Cognition, and Behaviour, Radboud University, Nijmegen - the Netherlands. (3) European Molecular Biology Laboratory (EMBL), Genomics Core Facility, Heidelberg - Germany

E-mail addresses (in the order of appearance): k.kole@neurophysiology.nl, y.komuro@neurophysiology.nl, jan.provaznik@embl.de, jelena.pistolic@embl.de, benes@embl.de, p.tiesinga@science.ru.nl, celikel@neurophysiology.nl (corresponding author)

Background (138)

Experience-dependent plasticity (EDP) is essential for anatomical and functional maturation of sensory circuits during development. Although the principal synaptic and circuit mechanisms of EDP are increasingly well studied experimentally and computationally, its molecular mechanisms remain largely elusive. EDP can be readily studied in the rodent barrel cortex, where each 'barrel column' preferentially represents deflections of its own principal whisker. Depriving select whiskers while sparing their neighbours introduces competition between barrel columns, ultimately leading to weakening of intracortical, translaminar (i.e. Cortical Layer (L)4-to-L2/3) feed-forward excitatory projections in the deprived columns. The same synapses are potentiated in the neighbouring spared columns. These experience-dependent alterations of synaptic strength are thought to underlie somatosensory map plasticity. We used RNA sequencing in this model system to uncover cortical-column and -layer specific changes on the transcriptome level that are induced by altered sensory experience.

Findings (66)

Column- and layer-specific barrel cortical tissues were collected from juvenile mice with all whiskers intact and mice that received 11-12 days long whisker (C-row) deprivation before high quality RNA was purified and sequenced. The current dataset entails an average of 50 million paired-end reads per sample, 75 base pairs in length. On average, 90.15% of reads could be uniquely mapped to the mm10 reference mouse genome.

Conclusions (32) – Word total for the abstract: 246 out of 250

The current data reveal the transcriptional changes in gene expression in the barrel cortex upon altered sensory experience in juvenile mice and will help to molecularly map the mechanisms of cortical plasticity.

Data Description

Context

Sensory experience powerfully shapes neural circuits. Changes due to sensory organ deprivation such as eye closure, digit amputation, and whisker trimming provide powerful means for studying mechanisms of experience dependent cortical plasticity.

In the whisker system experience dependent plasticity is most commonly studied in the barrel cortex subfield of the primary somatosensory cortex where neural representations of whiskers change in response to altered patterns of incoming sensory information. As originally shown in the barrel cortex [1] sensory deprivation induced by transient whisker trimming is sufficient to perturb neural receptive fields both during development and in adulthood. Previous work has also shown that the cellular basis of deprivation-induced decreases in whisker evoked representations are primarily due to a reduction of synaptic strength in monosynaptically connected feed-forward neuronal networks in behaving animals [2, 3]. Conversely whisker sparing induced enhancement in whisker representation is mediated at least in part by the long-term synaptic facilitation expressed along the L4 projections *in vivo* [4]. Identification of the molecular events that mediate these bidirectional changes in synaptic connectivity will benefit from systematic analysis of the gene transcription. Therefore, we performed RNA sequencing in the barrel cortex with or without sensory deprivation across cortical layers 2-4. This database will assist molecular and cellular neurobiologists in addressing the molecular mechanisms associated with experience dependent plasticity, and will enable statistical approaches to determine the dynamics of the coupled changes across molecular pathways as cortical circuits undergo plastic changes in their organization.

Methods

Animals

All experiments were performed in accordance with the Animal Ethics Committee of the Radboud University in Nijmegen, the Netherlands. Pregnant wild type mice (Charles River, stock number 000664) [RRID:NCBITaxon_10090] were kept at a 12-hour light/dark cycle with access to food *ad libitum*. Cages were checked for birth daily. To induce experience-dependent plasticity, pups underwent bilateral plucking of their C-row whiskers under isoflurane anaesthesia at P12 (**Figure 1**). Control animals were not plucked but anaesthetized and handled similarly. After recovery pups were returned to their home cage. Every other day pups were checked for whisker regrowth, which were plucked if present. At P23-P24, pups were randomly selected from their litter for slice preparation and tissue collection. For each experimental condition (i.e. whisker deprived or control), 4 female pups were used, thus each group consisted of 4 independent biological samples (also known as biological replicates). Samples from cortical layer (L) 4 and L2/3 were treated independently with their own corresponding groups of control, deprived, 1st order spared, 2nd order spared columns as detailed in Figure 1.

Figure 1 is about here

Slice preparation and sample collection

Pups were anaesthetized using isoflurane and then perfused with ice-cold carbogenated slicing medium (108 mM ChCl, 3 mM KCl, 26 mM $NaHCO_3$, 1.25 mM NaH_2PO_4, 25 mM glucose, 1 mM $CalCl_2$, 6 mM $MgSO_4$ and 3 mM Na-pyruvate). Next, pups were decapitated before the brain was quickly dissected out and 400 µm thalamocortical slices from each hemisphere were prepared as described before [2, 3]. Slices were transferred to 37 degrees Celsius carbogenated ACSF (120 mM NaCl, 3.5 mM KCl, 10 mM glucose, 2.5 mM $CaCl_2$, 1.3 mM $MgSO_4$, 25 mM $NaHCO_3$ and 1.25

mM NaH_2PO_4) where they were kept for 30 minutes and recovered at room temperature for another 30 minutes until tissue collection.

After incubation, slices were placed under a Nikon Eclipse FN1 microscope. The holding chamber was continuously perfused with room temperature carbogenated ACSF. Due to the 55 degree cut, slices were obtained in which S1 barrels from specific rows (A-E) could be identified [2]. A thin, long glass pipette was pulled using a Sutter instruments P-2000 pipette puller and was used to make intercolumnar incisions from L1 to the bottom of L4 after which the slice was placed under a binocular dissection microscope where the location of specific barrel columns could now be readily identified by eye. A sterile 32G needle was then used to cut out L2/3 and L4 separately from each column. Tissue from columns A/E and B/D were pooled as they both constitute second and first order spared whiskers, respectively. Immediately after dissection, tissue samples were snap frozen in liquid nitrogen and stored at -80 degrees Celsius until further use. All tools that came into direct contact with brain tissue were treated using RNAseZap in order to minimize RNAse contamination.

RNA isolation and quality control

Tissue samples originating from the same rows and layers were pooled within each animal. From control animals, only the C column tissues were used (also see *Re-use potential*). Tissue was quickly dissolved in Qiazol (Qiagen #79306), after which RNA was isolated using the miRNeasy Mini kit (Qiagen #217004), DNAse treated (Thermo Scientific, #EN0521) and cleaned up using RNeasy MinElute kit (Qiagen #74204), all following the manufacturer's instructions. Samples were then stored at -80 degrees Celsius until further processing.

RNA sample integrity was determined using Agilent Tapestation (High Sensitivity RNA Screentape). Sample RINs ranged from 7.1 to 8.8. To further assess RNA purity and integrity,

RNA samples were used in RT-PCR to confirm that cDNA could be produced and that a large (~1000 bp) amplicon could be obtained. To produce cDNA, SuperScript® II Reverse Transcriptase (Thermo Scientific #18064014) and random hexamer primers (Roche #11034731001) were used. The resulting cDNA was then added to a PCR reaction mix which further consisted of Jumpstart Ready Mix (Sigma P2893) and exon-exon junction spanning CamKII primers (FW TCCAACATTGTACGCCTCCAT; RV TGTTGGTGCTGTCGGAAGAT). From all cDNA samples a fragment of the expected size could be amplified, suggesting that the RNA samples contained pure RNA of sufficient integrity. All RNA samples thus passed our quality control criteria and were subjected to RNA sequencing.

RNA sequencing

RNA sequencing was conducted at the Genomics Core Facility of the EMBL, Heidelberg, Germany [RRID:SCR_004473]. The cDNA library was generated using the non-stranded NEBNext Ultra RNA Library Preparation Kit for Illumina (New England Biolabs, catalogue #E7530), which includes oligo-dT bead selection of mRNA. For library enrichment, 13-14 PCR cycles were performed. Pooled libraries were sequenced on the Illumina NextSeq 500 instrument [RRID:SCR_014983] in a 75bp paired-end mode using High output flow cells.

Data validation and quality control

Sequencing read quality was assessed using FastQC (Babraham Bioinformatics) [RRID:SCR_014583], the results of which were merged using MultiQC (http://multiqc.info) [RRID:SCR_014982]. Results are displayed in **Figure 2**. Per base quality *phred* scores range from 34.80 to 35.15, indicating base call accuracies of >99.9% (**Figure 2A**). Overall 91.48-94.03% of reads had a mean *phred* score of 30 or above (**Figure 2B**). In line with these scores, per base N content (i.e. percentage of bases that could not confidently called) was very low, with a maximum value 0.053%.

Figure 2 is about here

Reads were then mapped to the mm10 reference genome using STAR [5] [RRID:SCR_005622], which uniquely mapped between 39,000,000 and 59,000,000 reads, constituting an average 90.15% unique map rate across samples (**Figure 2D**). Since the library preparation protocol entails a PCR enrichment step, which can lead to technical duplication and hence an overestimation of observed transcripts, we used Seqmonk (Babraham Bioinformatics) [RRID:SCR_001913] to plot the read density against the duplication levels (i.e. the percentage of duplicate reads) for each transcript. The obtained duplication plots showed a clear positive relation between read density and duplication levels (**Figure 3** and **Supplemental Figure 1**), suggesting that the origin of read duplication is biological, rather than technical.

Based on the above quality control measures we determined that our RNA-sequencing data was of sufficient quality to be used in downstream analyses, therefore we continued with gene expression analysis.

Figure 3 is about here

Analysis of gene expression

Using a 2 read cut-off we identified 16,900 to 17,600 transcripts per sample (**Figure 4A**). Raw gene counts can be found online (see Supporting Data – DOI to appear). Differential gene expression analyses across groups were performed using EdgeR v3.12.1 [6, 7] [RRID:SCR_012802] using only genes with a count per million (CPM) >1 in at least 4 samples (**Supplementary Table 1** for details on the commands used). Since laminar identity is an important feature of our experimental setup, we assessed the relative expression of known

molecular markers for L2/3 (*Cacna1h*, *Id2*, *Igfbp4*, *Igfn1*, *Mdga1*, *Plcxd1*, *Rasgrf2*, *Rgs8*, *Tle3*) and L4 (*Cartpt*, *Cyp39a1*, *Kcnh5*, *Kcnip2*, *Lmo3*, *Rorb*, *Scnn1a*) [8–10], which showed selective enrichment of the laminar markers in isolated layers (**Figure 4B**).

Figure 4 is about here

To assess the variance in transcript counts, we calculated the coefficient of variation (CV) for each transcript with a cut-off of 50 as the minimal read count separately for each group (**Figure 4C**). This analysis showed that, on average, 85.93% of transcripts have a CV below 15%, suggesting low variance across transcript counts for individual genes. Principal component analysis (PCA) showed that samples cluster based on layer, and the first two components explained ~88% variance the data (**Figure 4C**, **Supplemental Figure 2B**).

These quality control routines suggest that we have obtained RNA-sequencing data of high read quality, with individual bases being called confidently throughout the length of reads, which uniquely map to the mm10 reference genome at high rates (>90% average). The laminar origin of our samples could be identified through known molecular markers, confirming our samples are of high anatomical specificity.

Re-use potential

The current RNA-seq dataset might help address the molecular underpinnings of cortical experience-dependent plasticity. For example, it could be used (1) to identify genes whose transcription is modulated in an experience-dependent manner, (2) to statistically map the transcriptional networks at laminar resolution, (3) creating synergy with the single neuron RNA-seq datasets [11, 12], to address the molecular diversity of the cortical networks, (4) combined with the proteomic analysis performed under comparable experimental conditions in the

accompanying manuscript (Kole et al, submitted), to systematically study the transcriptional and translational regulation of the genome upon altered sensory experience, and finally (5) to identify and quantify splice isoforms, given the sequencing depth of the current dataset. Since splicing and other posttranscriptional mechanisms govern which proteins are ultimately produced, combining the current transcriptomic dataset with a proteomics approach would also be of high importance.

The current dataset focuses on isolated cortical columns and layers, which are necessarily diverse samples containing neuronal and non-neuronal cell classes. In terms of experience dependent plasticity, although most previous studies focus on excitatory projections, inhibitory cells and even non-neuronal cells have been implicated in plasticity [13–15]. This heterogeneity might be particularly important for L2/3, as also shown by the principal component analysis (**Figure 4D**), given the relative diversity of cellular populations in supragranular layers and their heterogeneous connectivity patterns [16].

Researchers reusing our dataset should be aware that comparisons between control column C and spared columns (A/E, B/D) may have to be approached with caution, as this would involve two different columnar identities (whose transcriptomic dissimilarities are currently unknown), each coming from cortices that have had different sensory experience. However direct comparisons between the C columns across experimental conditions (i.e control versus deprived) as well as within-animal across-column comparisons in deprived animals control for these confounding variables.

Taken together we hope that this data will prove useful in discovering novel molecular targets responsible for cortical plasticity and will lead to targeted control of plasticity in health and disease.

References

1. Hand PJ (1892) Plasticity of the rat cortical barrel system. In: Strick P, Morrison AD (eds) Changing concepts of the nervous system. Academic Press, New York, pp 49–75
2. Allen CB, Celikel T, Feldman DE (2003) Long-term depression induced by sensory deprivation during cortical map plasticity in vivo. Nat Neurosci 6:291–9. doi: 10.1038/nn1012
3. Celikel T, Szostak VA, Feldman DE (2004) Modulation of spike timing by sensory deprivation during induction of cortical map plasticity. Nat Neurosci 7:534–41. doi: 10.1038/nn1222
4. Clem RL, Celikel T, Barth AL (2008) Ongoing in vivo experience triggers synaptic metaplasticity in the neocortex. Science 319:101–4. doi: 10.1126/science.1143808
5. Dobin A, Davis CA, Schlesinger F, Drenkow J, Zaleski C, Jha S, Batut P, Chaisson M, Gingeras TR (2013) STAR: ultrafast universal RNA-seq aligner. Bioinformatics 29:15. doi: 10.1093/bioinformatics/bts635
6. Robinson MD, McCarthy DJ, Smyth GK (2010) edgeR: a Bioconductor package for differential expression analysis of digital gene expression data. Bioinformatics 26:139. doi: 10.1093/bioinformatics/btp616
7. McCarthy DJ, Chen Y, Smyth GK (2012) Differential expression analysis of multifactor RNA-Seq experiments with respect to biological variation. Nucleic Acids Res 40:4288. doi: 10.1093/nar/gks042
8. Molyneaux BJ, Goff LA, Rinn JL, Arlotta P (2015) Genome-wide Analysis of In Vivo Transcriptional Dynamics during Pyramidal Neuron Fate Selection in Neocortex NeuroResource DeCoN : Genome-wide Analysis of In Vivo Transcriptional Dynamics during Pyramidal Neuron Fate Selection in Neocortex. 275–288.
9. Xue M, Atallah B V., Scanziani M (2014) Equalizing excitation–inhibition ratios across visual cortical neurons. Nature 511:596–600. doi: 10.1038/nature13321
10. Rowell JJ, Mallik AK, Dugas-Ford J, Ragsdale CW (2010) Molecular analysis of neocortical layer structure in the ferret. J Comp Neurol 518:3272–3289. doi: 10.1002/cne.22399
11. Zeisel A, Munoz-Manchado AB, Codeluppi S, Lonnerberg P, La Manno G, Jureus A, Marques S, Munguba H, He L, Betsholtz C, Rolny C, Castelo-Branco G, Hjerling-Leffler J, Linnarsson S (2015) Cell types in the mouse cortex and hippocampus revealed by single-cell RNA-seq. Science (80-) 347:1138–1142. doi: 10.1126/science.aaa1934
12. Tasic B, Menon V, Nguyen TN, Kim TK, Jarsky T, Yao Z, Levi B, Gray LT, Sorensen SA, Dolbeare T, Bertagnolli D, Goldy J, Shapovalova N, Parry S, Lee C, Smith K, Bernard A, Madisen L, Sunkin SM, Hawrylycz M, Koch C, Zeng H (2016) Adult mouse cortical cell taxonomy revealed by single cell transcriptomics. Nat Neurosci 19:335–346. doi: 10.1038/nn.4216
13. Tropea D, Van Wart A, Sur M (2009) Molecular mechanisms of experience-dependent plasticity in visual cortex. Philos Trans R Soc Lond B Biol Sci 364:341–55. doi: 10.1098/rstb.2008.0269
14. Kole K (2015) Experience-dependent plasticity of neurovascularization. J Neurophysiol 114:2077–9. doi: 10.1152/jn.00972.2014
15. Foeller E, Celikel T, Feldman DE (2005) Inhibitory sharpening of receptive fields contributes to whisker map plasticity in rat somatosensory cortex. J Neurophysiol 94:4387–400. doi: 10.1152/jn.00553.2005
16. Markram H, Muller E, Ramaswamy S, Reimann MW, Abdellah M, Sanchez CA, Ailamaki A, Alonso-Nanclares L, Antille N, Arsever S, Kahou GAA, Berger TK, Bilgili A, Buncic N, Chalimourda A, Chindemi G, Courcol J-D, Delalondre F, Delattre V, Druckmann S, Dumusc R, Dynes J, Eilemann S, Gal E, Gevaert ME, Ghobril J-P, Gidon A, Graham JW, Gupta A, Haenel V, Hay E, Heinis T, Hernando JB, Hines M, Kanari L, Keller D, Kenyon

J, Khazen G, Kim Y, King JG, Kisvarday Z, Kumbhar P, Lasserre S, Le Bé J-V, Magalhães BRC, Merchán-Pérez A, Meystre J, Morrice BR, Muller J, Muñoz-Céspedes A, Muralidhar S, Muthurasa K, Nachbaur D, Newton TH, Nolte M, Ovcharenko A, Palacios J, Pastor L, Perin R, Ranjan R, Riachi I, Rodríguez J-R, Riquelme JL, Rössert C, Sfyrakis K, Shi Y, Shillcock JC, Silberberg G, Silva R, Tauheed F, Telefont M, Toledo-Rodriguez M, Tränkler T, Van Geit W, Díaz JV, Walker R, Wang Y, Zaninetta SM, DeFelipe J, Hill SL, Segev I, Schürmann F (2015) Reconstruction and Simulation of Neocortical Microcircuitry. Cell 163:456–492. doi: 10.1016/j.cell.2015.09.029

Availability of the supporting data

Supporting data are available online (https://goo.gl/tBof51) and will be distributed via GigaScience DB.

Raw sequence reads were deposited in NCBI GEO.

Link: https://www.ncbi.nlm.nih.gov/geo/query/acc.cgi?acc=GSE90929

List of abbreviations

EDP	Experience dependent plasticity
L2/3	Cortical Layer 2/3, also known as supragranular layers
L4	Cortical Layer 4, i.e. granular layer

Competing interests

The authors declare no competing interests.

Author contributions

KK performed all experimental manipulations, sample acquisition, biological and bioinformatic quality controls, and prepared the tables and figures. YK and JaP performed bioinformatic analysis. JeP performed library prep. VB supervised RNA-seq. PT contributed bioinformatic analysis and co-supervised the project. TC designed and supervised the project. KK and TC wrote the manuscript. All authors edited otherwise approved the final version of the manuscript.

Figure Legends

Figure 1. Overview of the experimental design, sample collection and data organization. **(A)** Pups were bilaterally spared or deprived of off their C-row whiskers between P12 and P23-P24, when acute slices are made and column- and layer-specific tissues were excised. **(B)** RNA was isolated, checked for integrity and purity, and subsequently sequenced. **(C)** Organization of the database. Colour codes denote experimental groups. Same denominations are used in the read counts matrix file (see **Supplemental Data**).

Figure 2. FastQC and STAR output graphs for all samples. **(A-B)** *Phred* scores per base and per sequence. **(C)** Per sequence GC content. **(D)** STAR output of alignment scores.

Figure 3. Overlays of duplication plot contours, showing a positive correlation between read density and duplication levels. Depicted contours enclose 90% of the data points.

Figure 4. Gene expression analyses. **(A)** Histogram of read counts per transcript per sample. With a cut-off of 2 reads, between 16,900 and 17,600 transcripts could be identified across samples. **(B)** Relative expression of known molecular markers for cortical laminae. Layer 4 markers are enriched in samples originating from this layer; the same is true for Layer 2/3 marker expression in Layer 2/3 samples. **(C)** Cumulative plots of the coefficient of variance (CV) of individual experimental groups. Including only transcripts identified by 50 reads or more, average CVs of <15% are found in ~85% of transcripts. **(D)** Principal component analysis (PCA) showing sample clustering by layer, including only transcripts identified by at least 50 reads. Principal component (PC) 1 and 2 account for 88% of overall variance.

Supplemental Figure 1. Duplication plots for all samples, produced using SeqMonk (Babraham Bioinformatics).

Supplemental Figure 2. (A) Cumulative plots of the coefficient of variance (CV) of experimental each group, including transcripts identified by at least one read. Average CVs of <25% are found in ~85% of transcripts. **(B)** Principal component analysis (PCA) including transcripts identified by at least one read. The majority (88%) of overall variance is explained by Principal components (PC) 1 and 2.

Figure1
Click here to download Figure Figure1.pdf

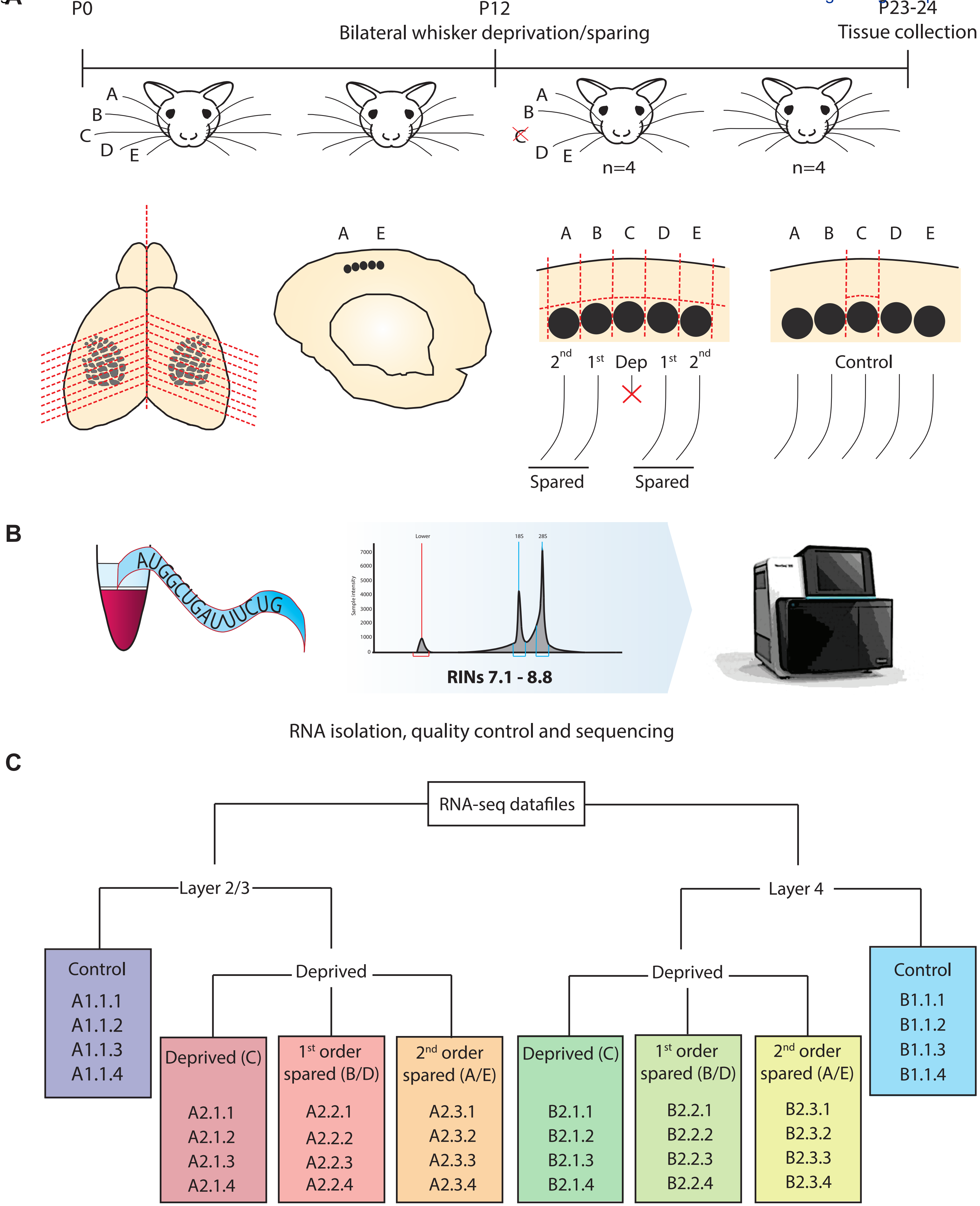

Figure2

Click here to download Figure Figure2.pdf

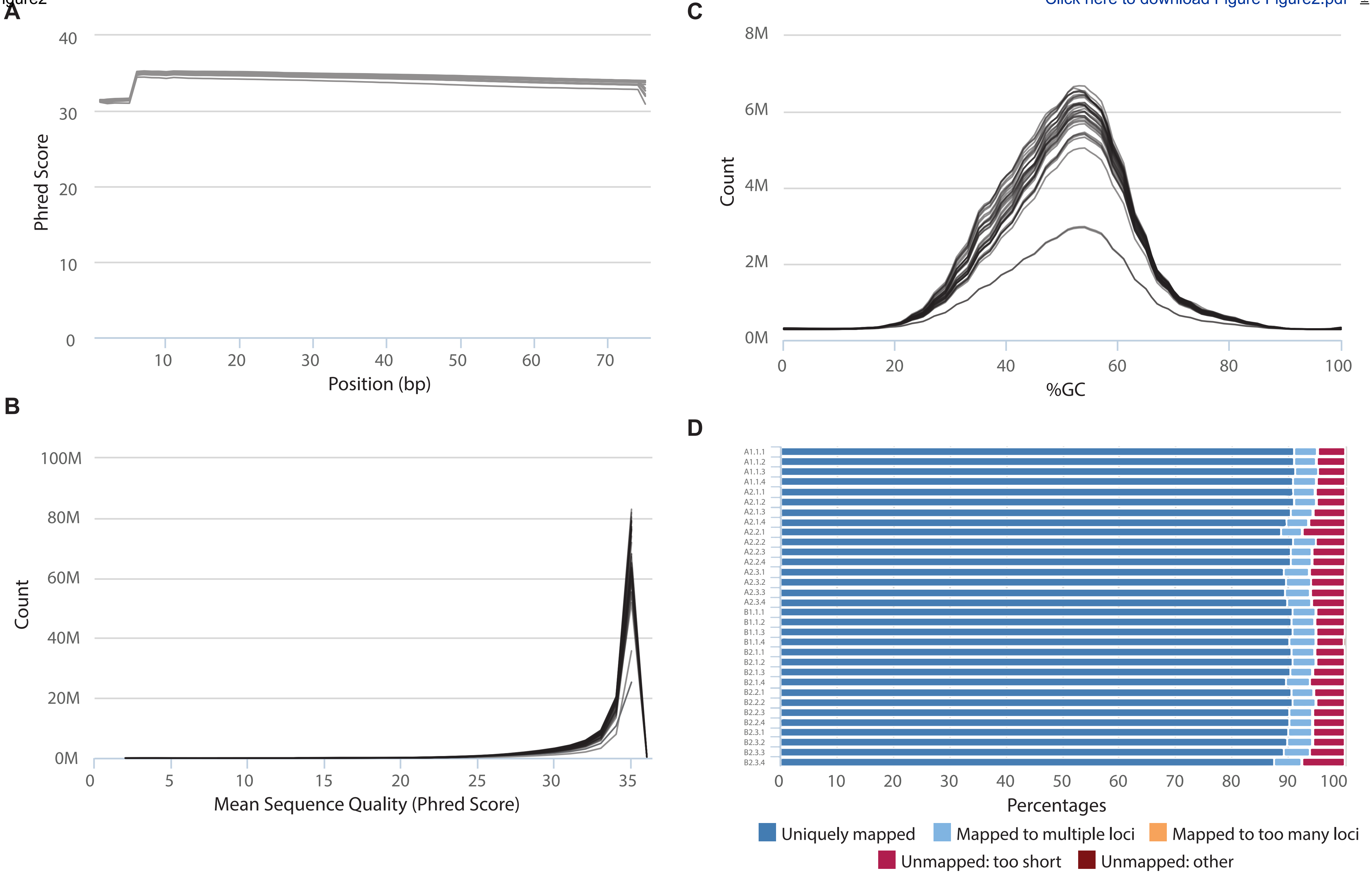

Figure3

Click here to download Figure Figure3.pdf

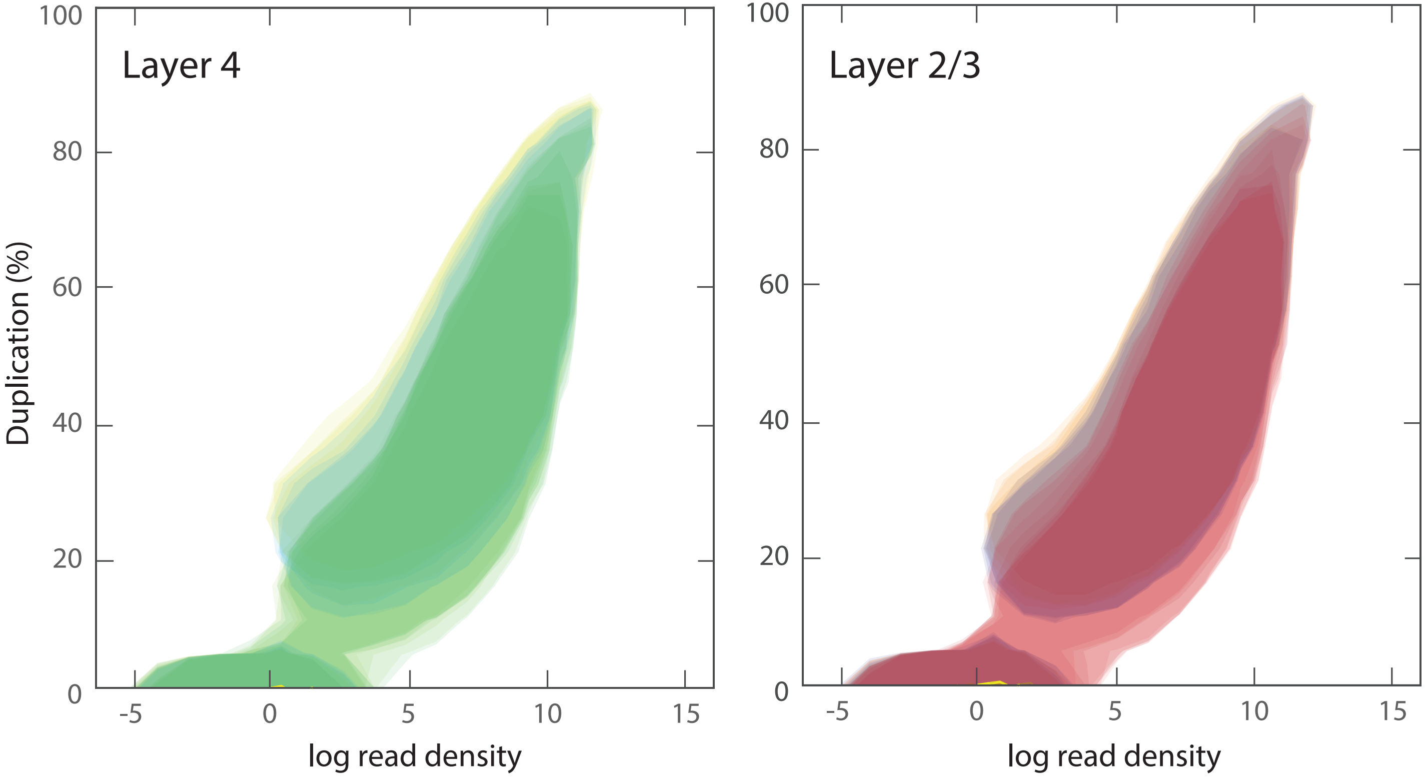

Figure4

Click here to download Figure Figure_4.pdf

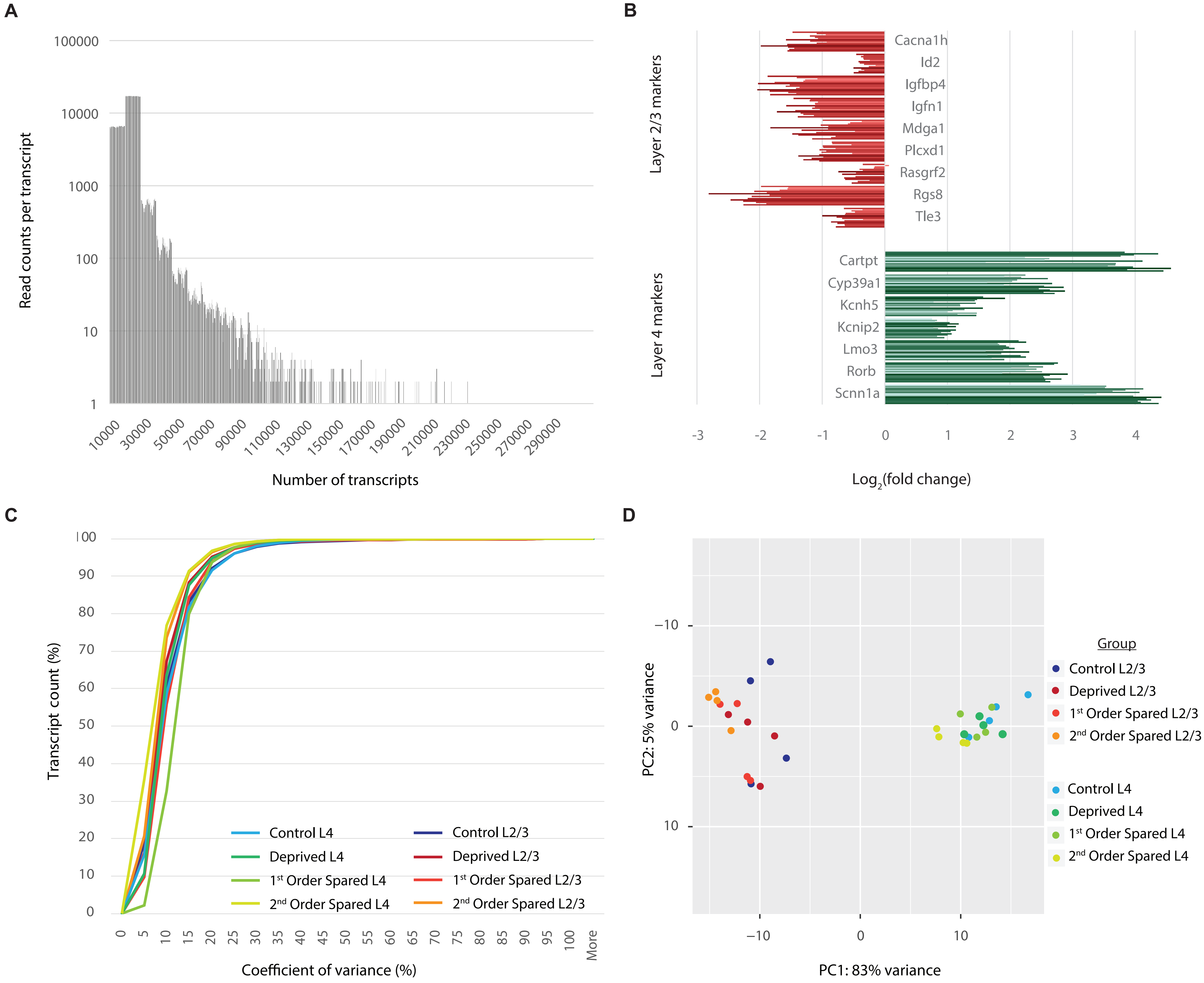

Supplementary Figure 1

Click here to access/download
Supplementary Material
SupplementalFigure1.pdf

Supplementary Figure 2

Click here to access/download
Supplementary Material
SupplementalFigure2.pdf

Supplementary Table 1

Click here to access/download
Supplementary Material
Supplementary_Table1_EdgeRcommands.txt

Revised manuscript, Word .docx version

Click here to access/download
Supplementary Material
Gigascience_Kole_RevisedRound2.docx
