# Supplementary figures and images for: Transcriptional mapping of the primary somatosensory cortex upon sensory deprivation
Source: Gigascience. 2017 Aug 23;6(10):1–6. doi: 10.1093/gigascience/gix081 (PMC5965344; doi:10.1093/gigascience/gix081)

Control C L2/3

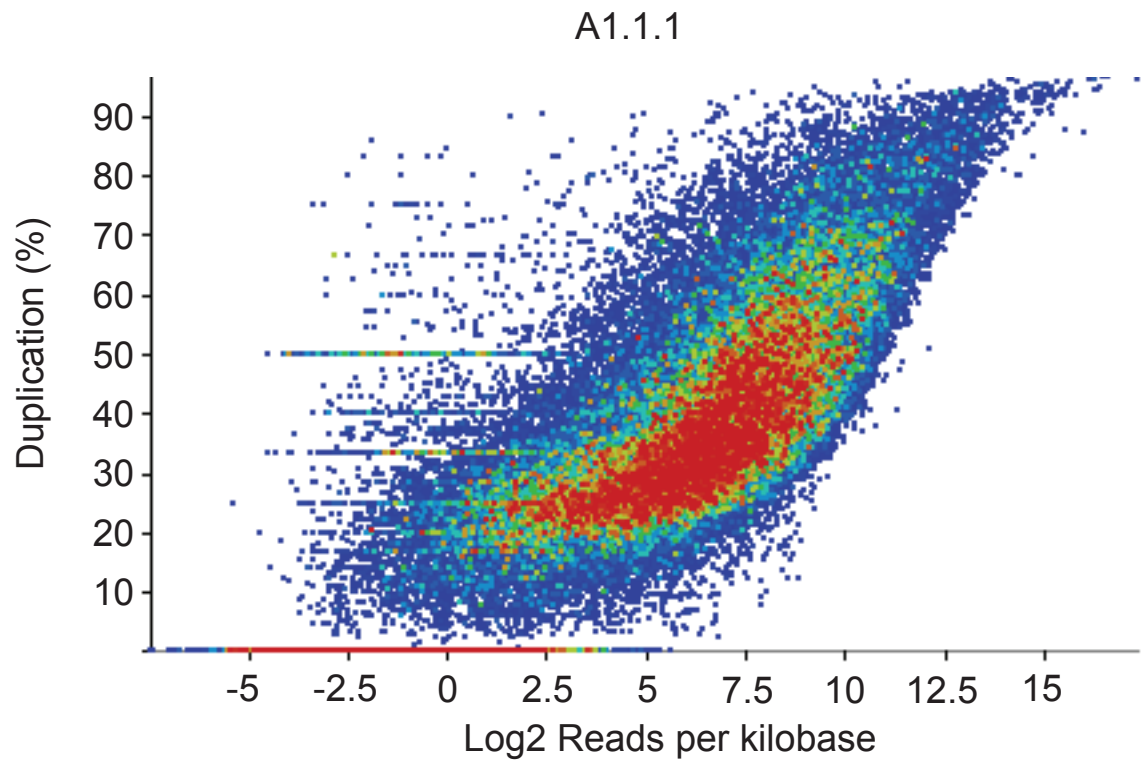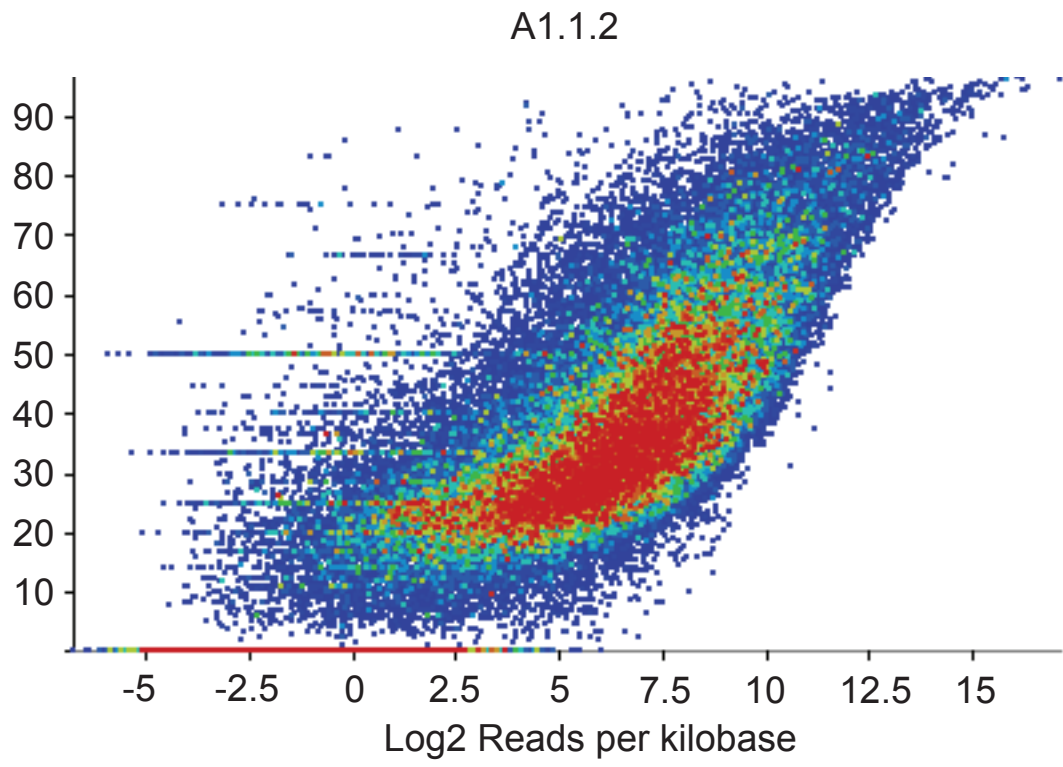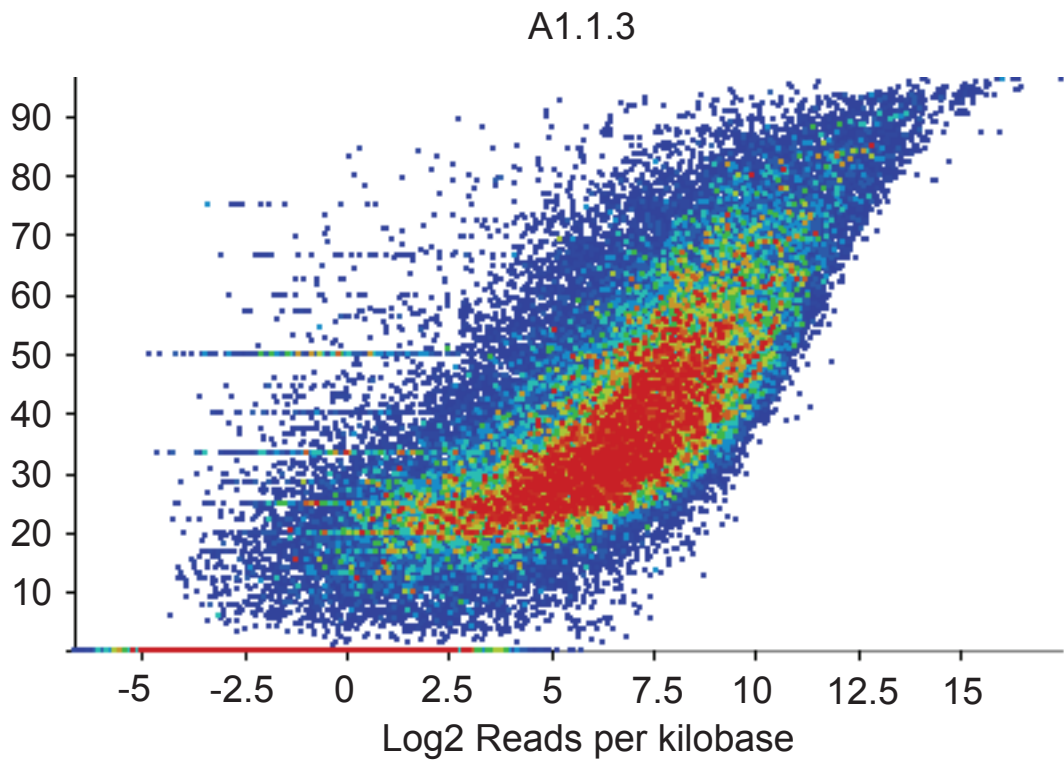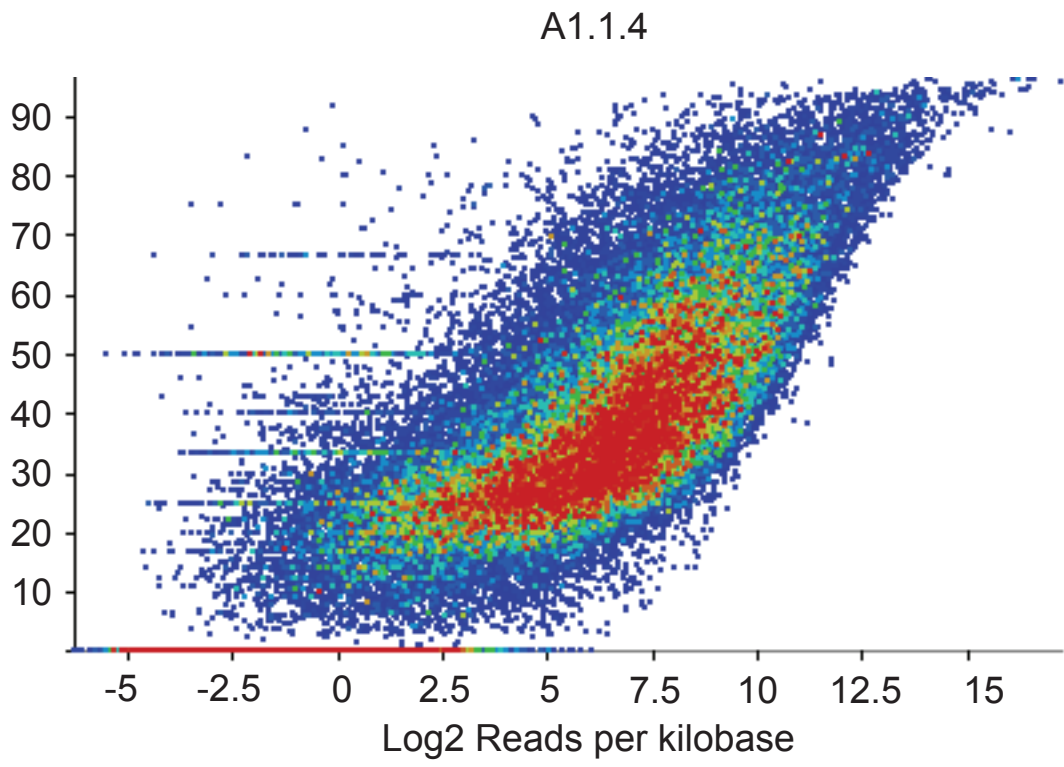

Control C L4

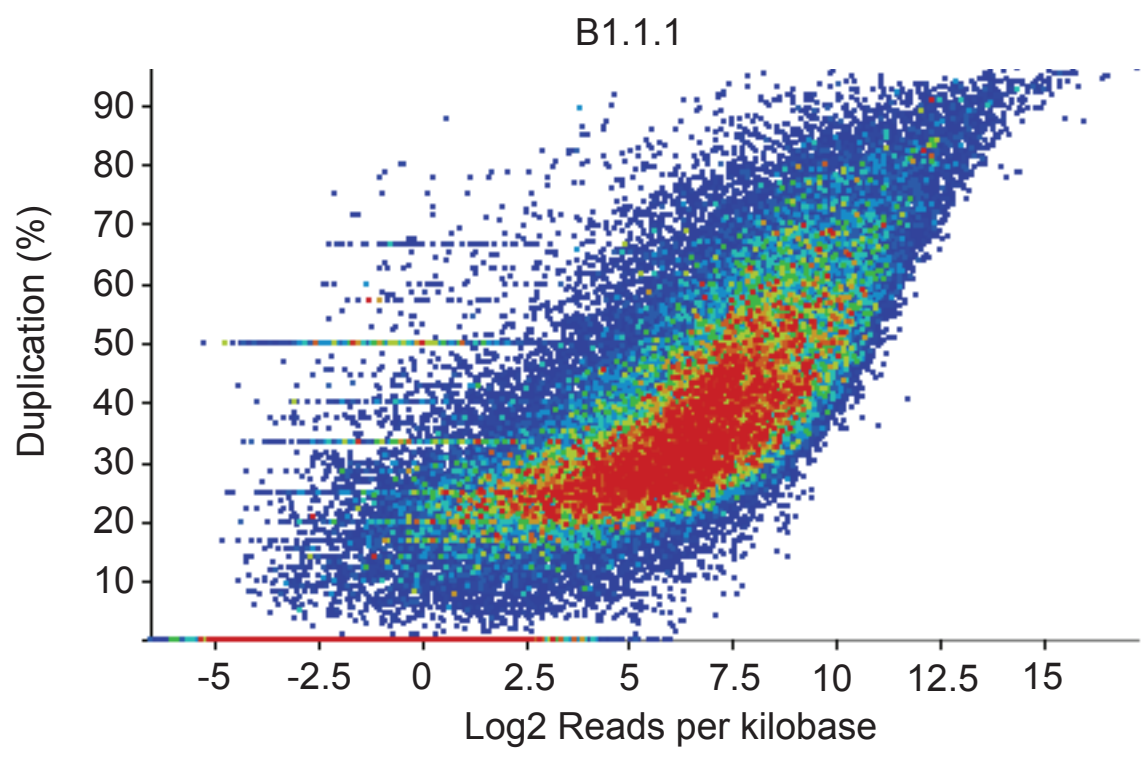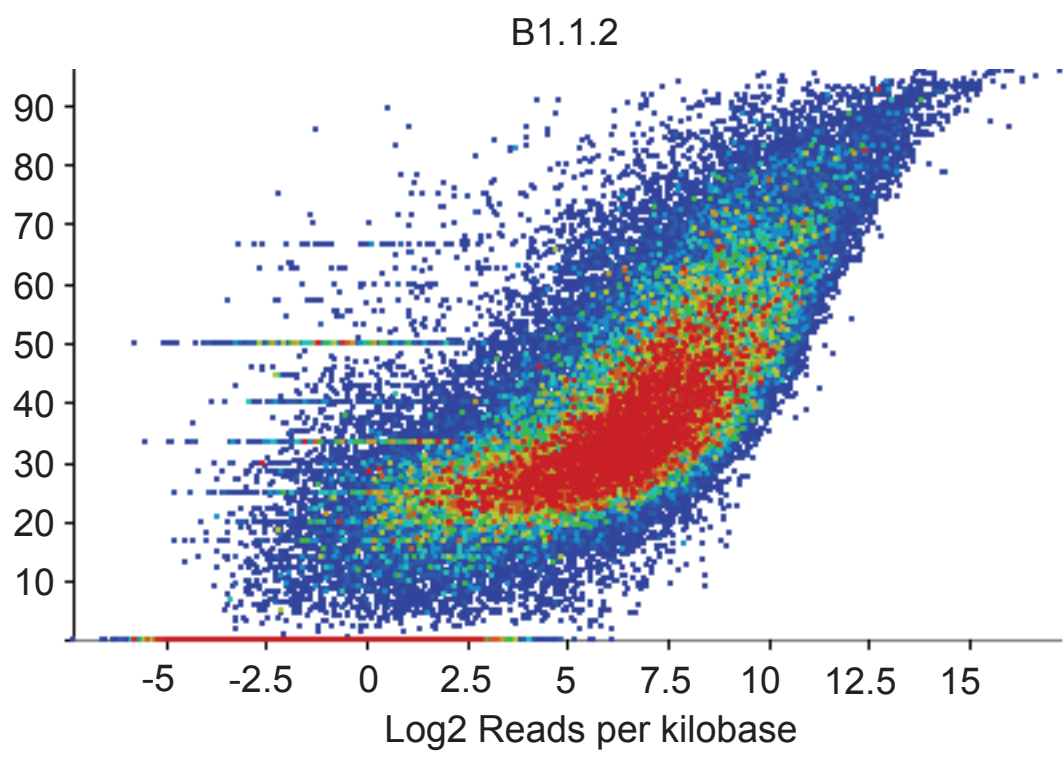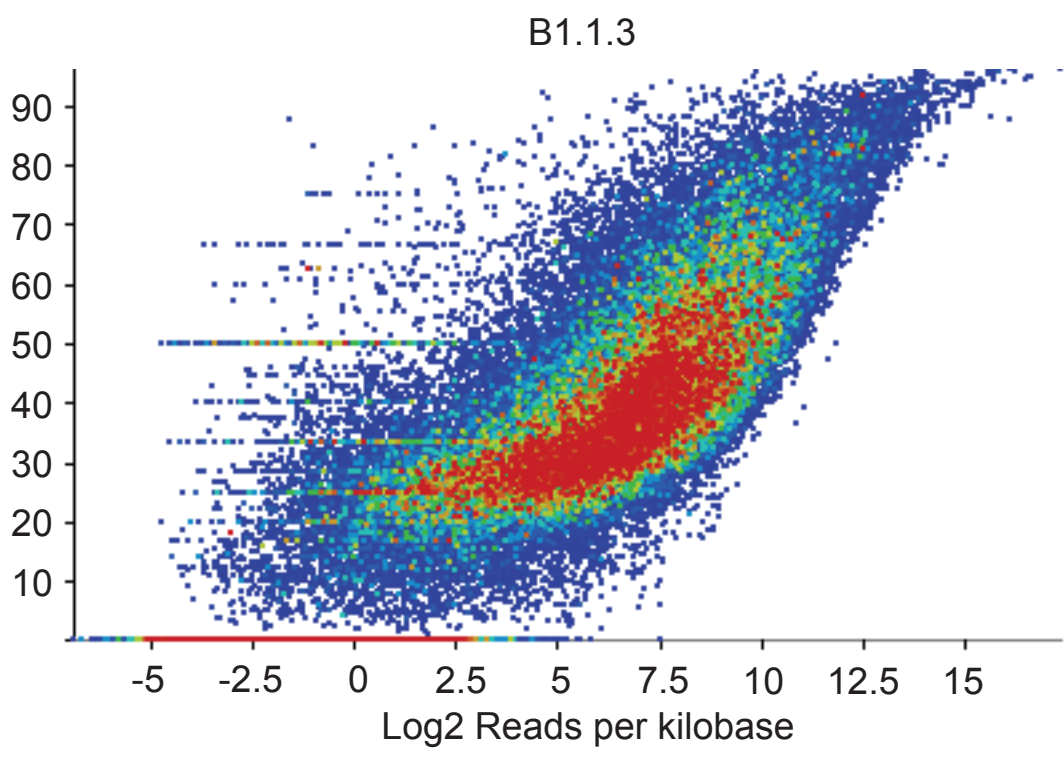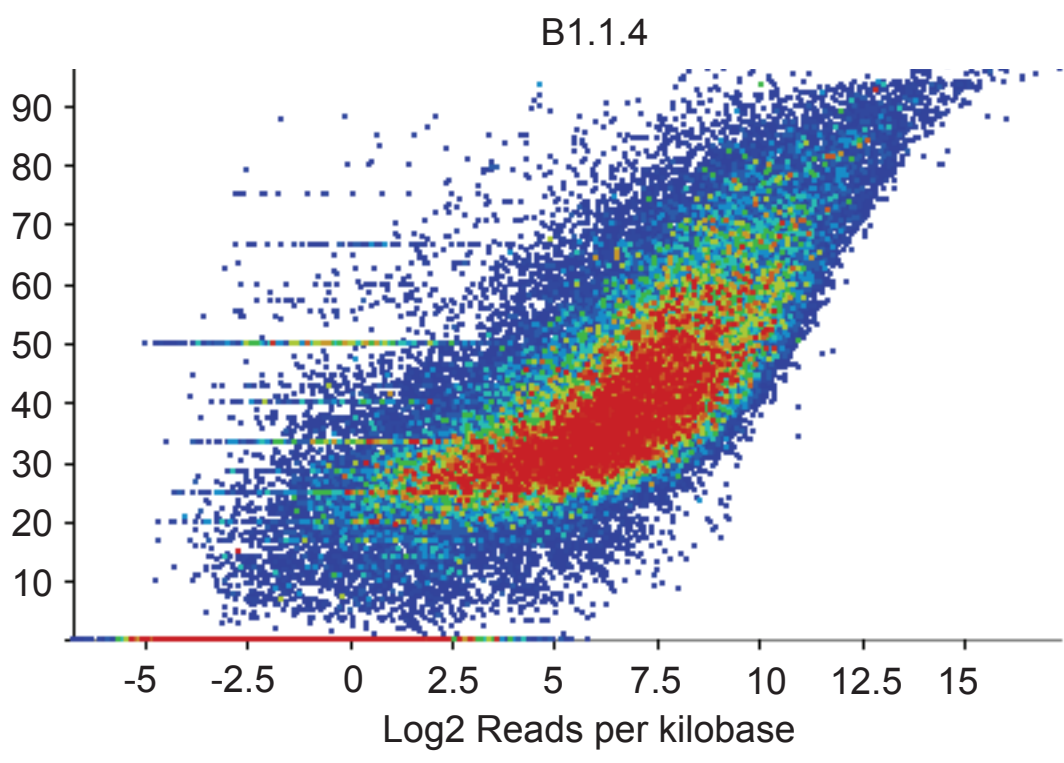

Deprived C L2/3

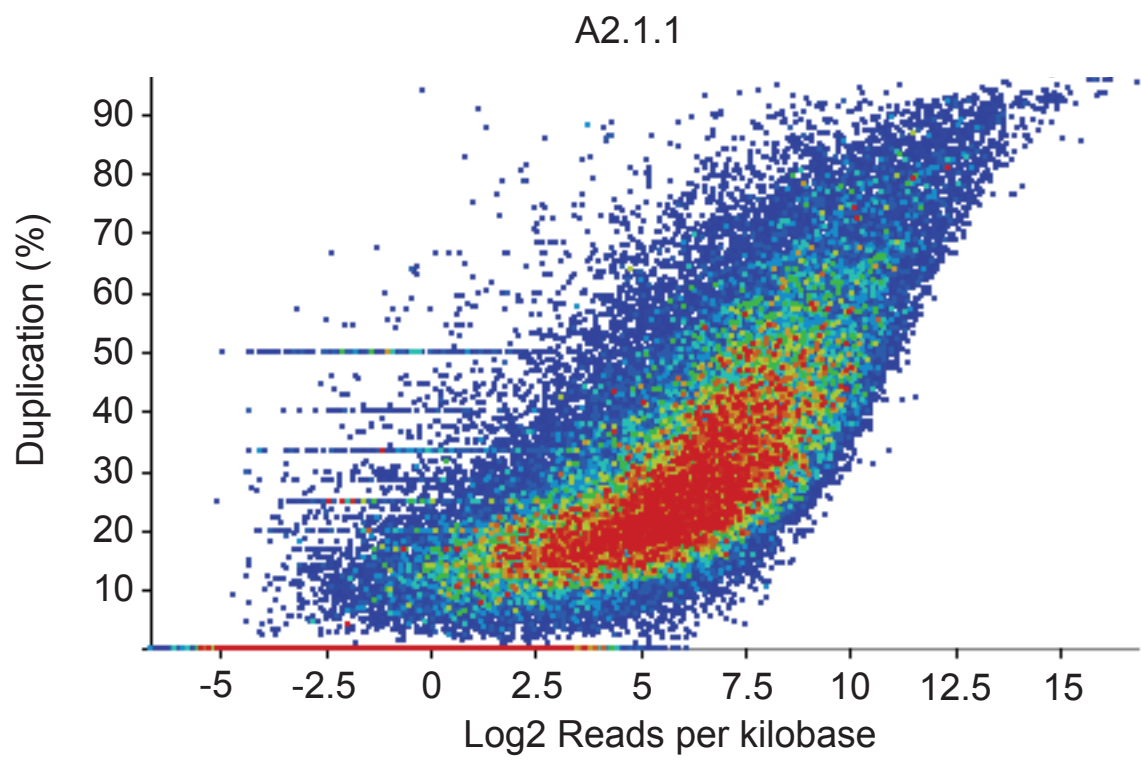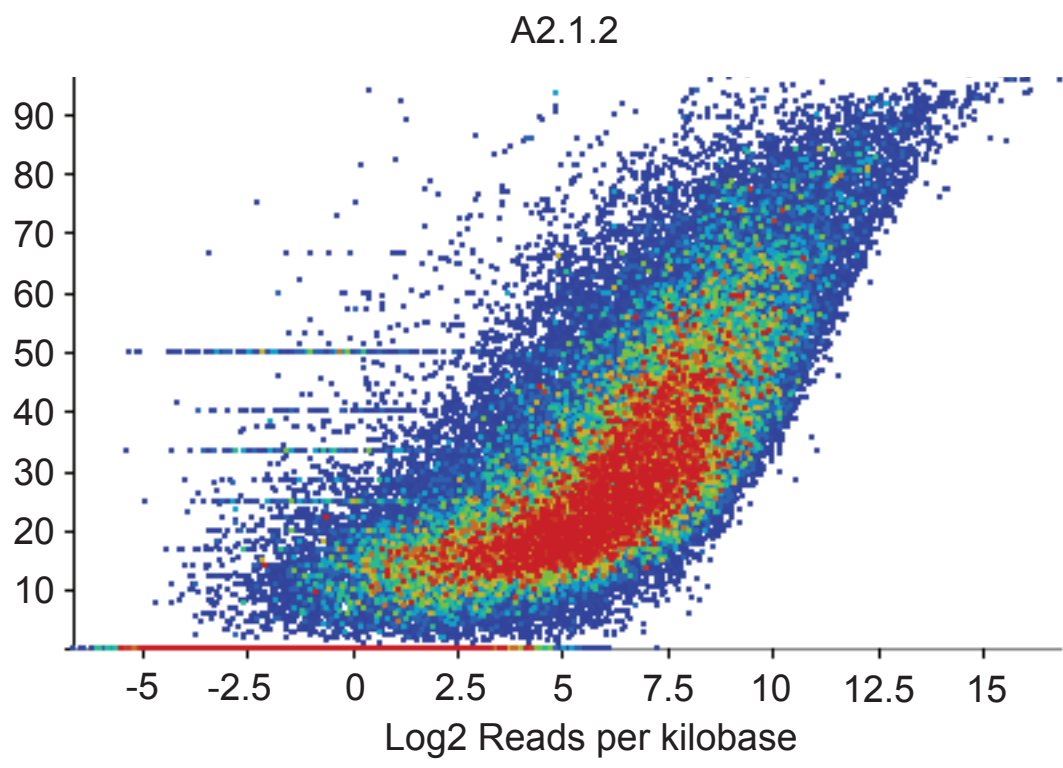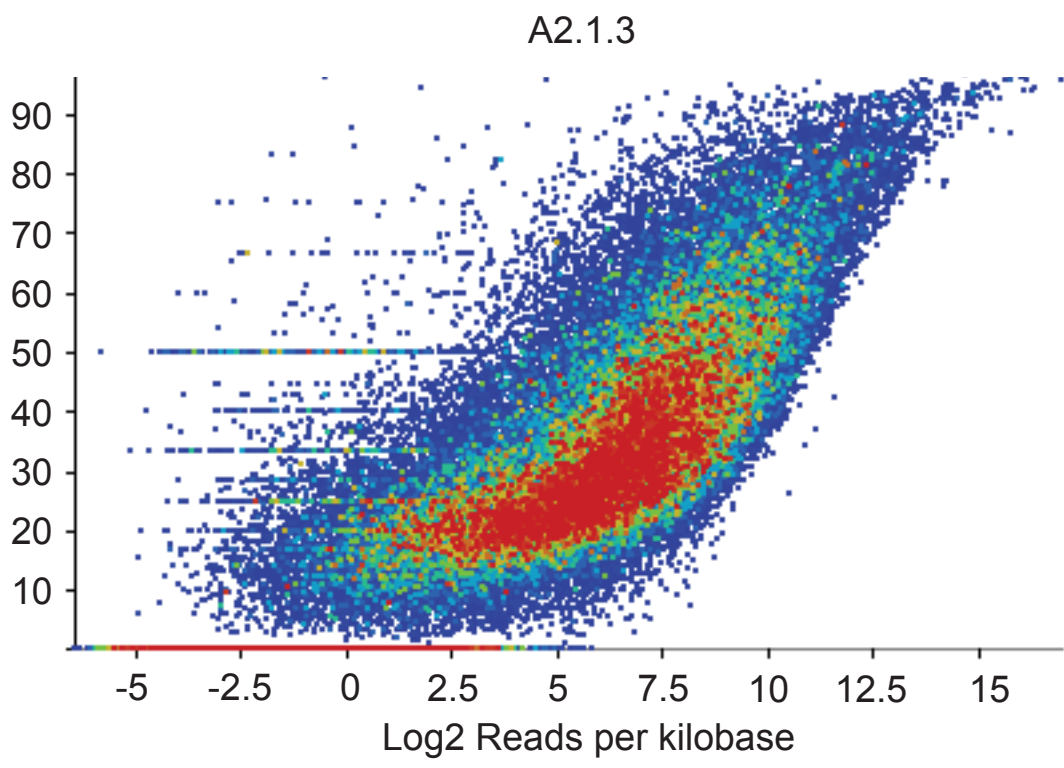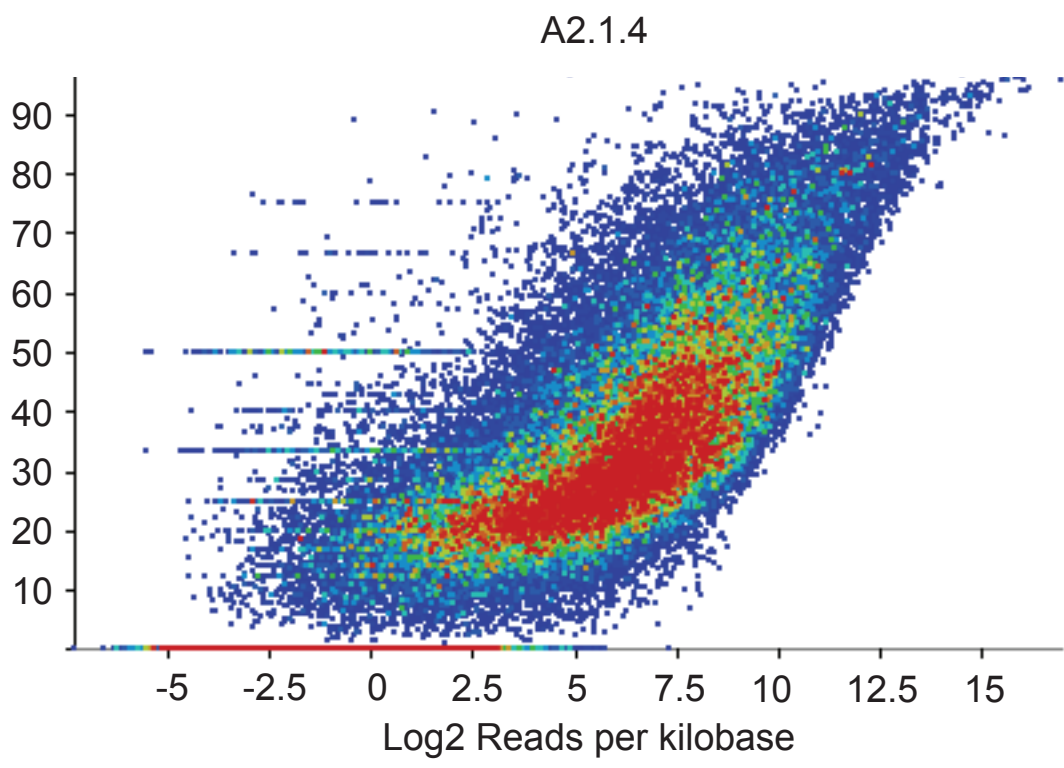

Deprived C L4

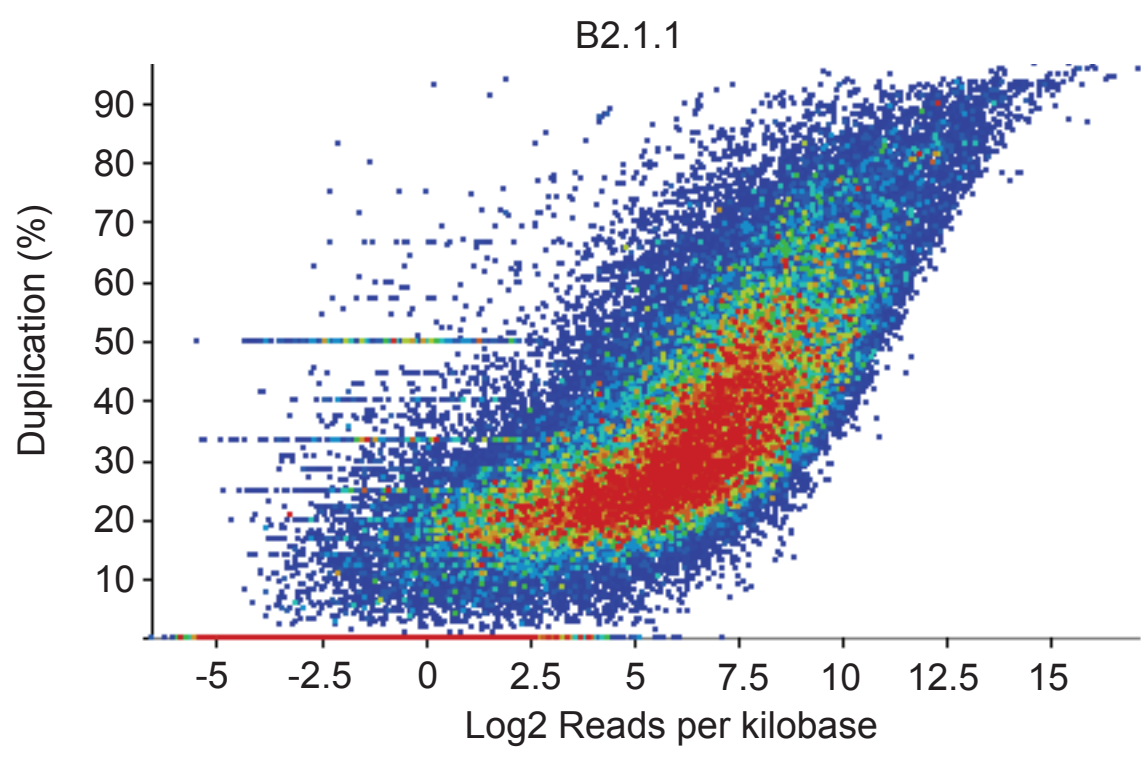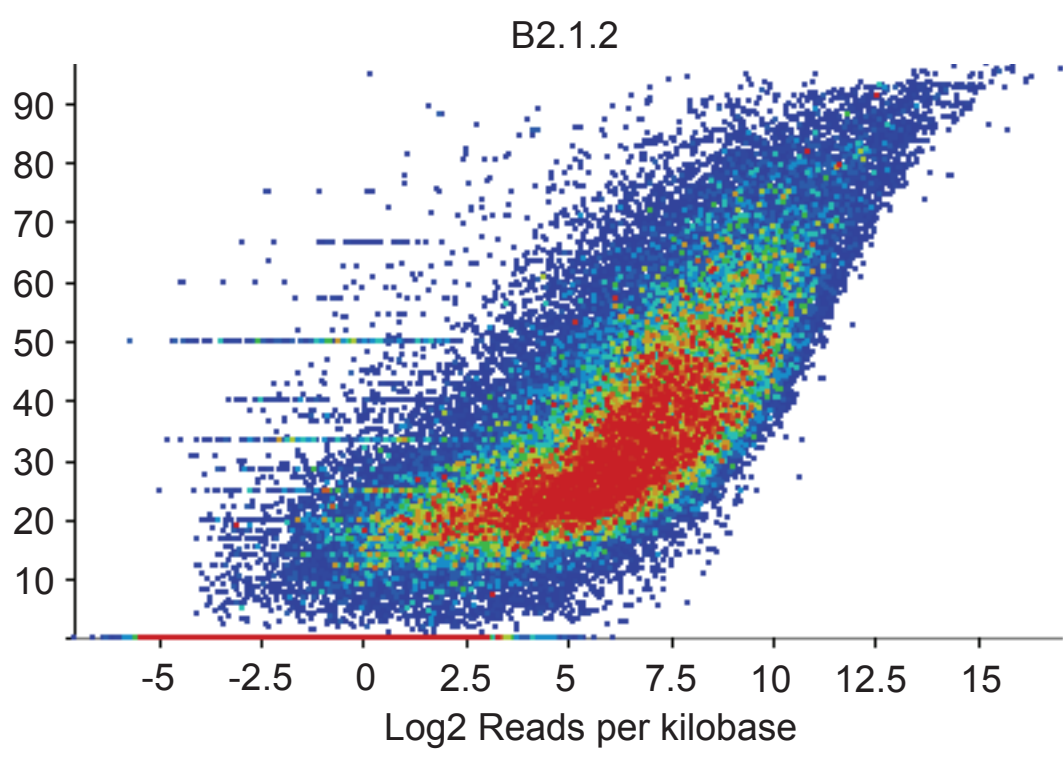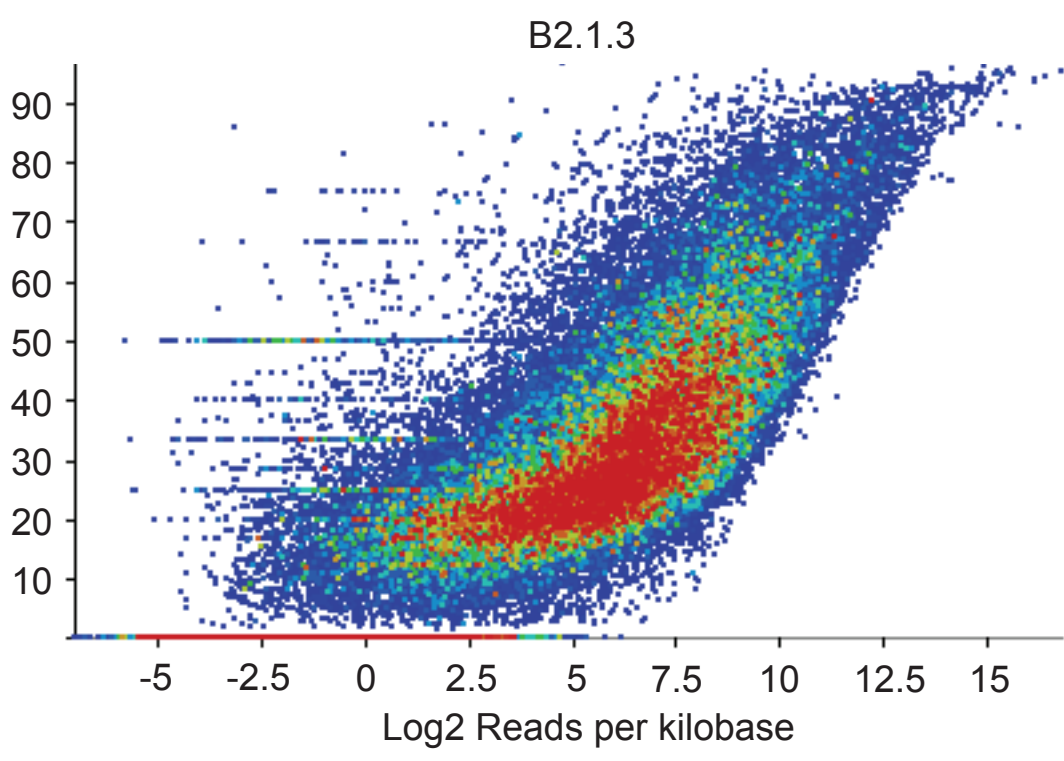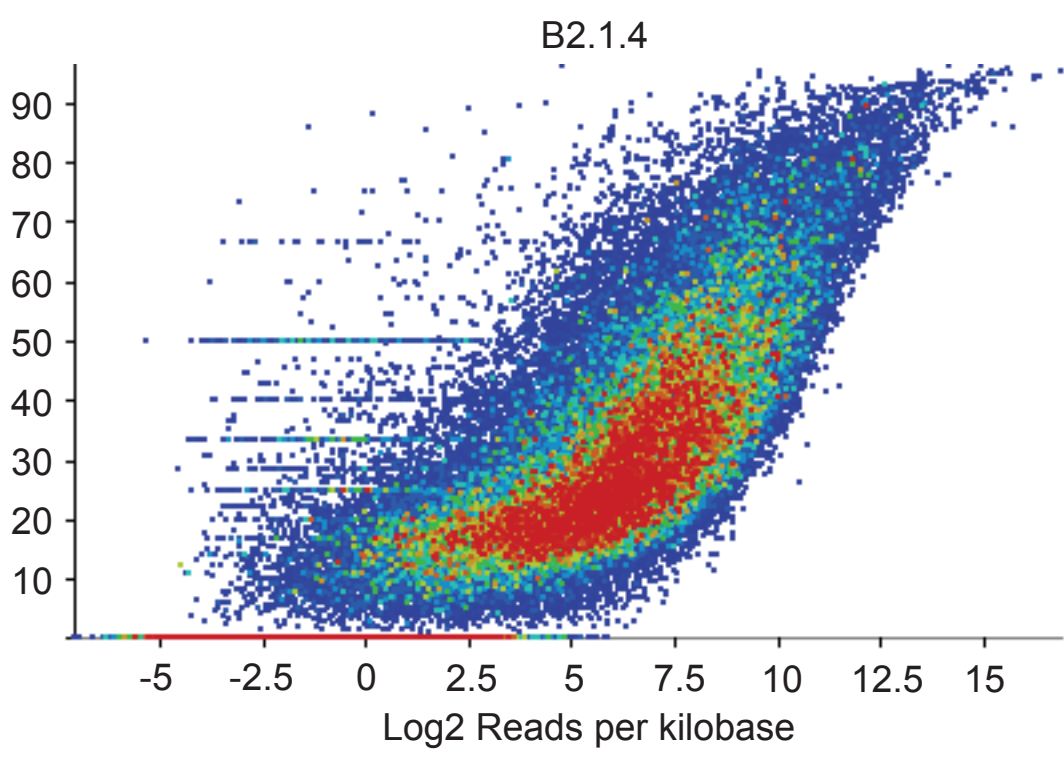

Spared B/D L2/3

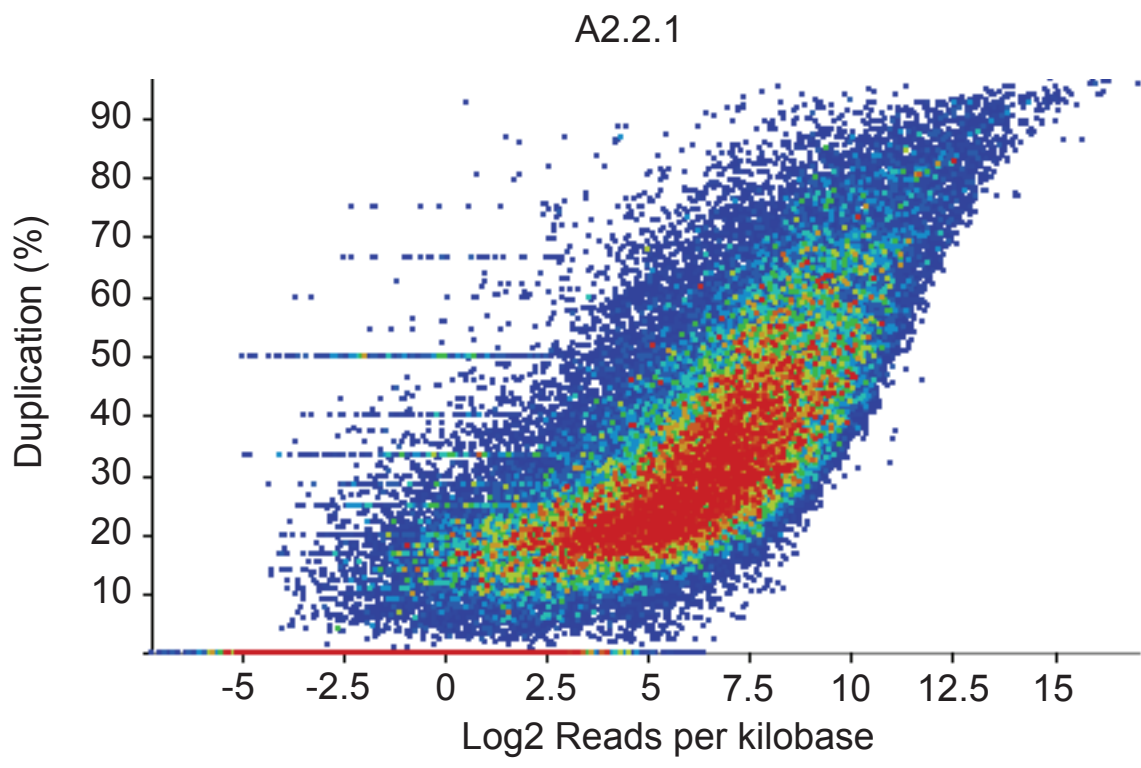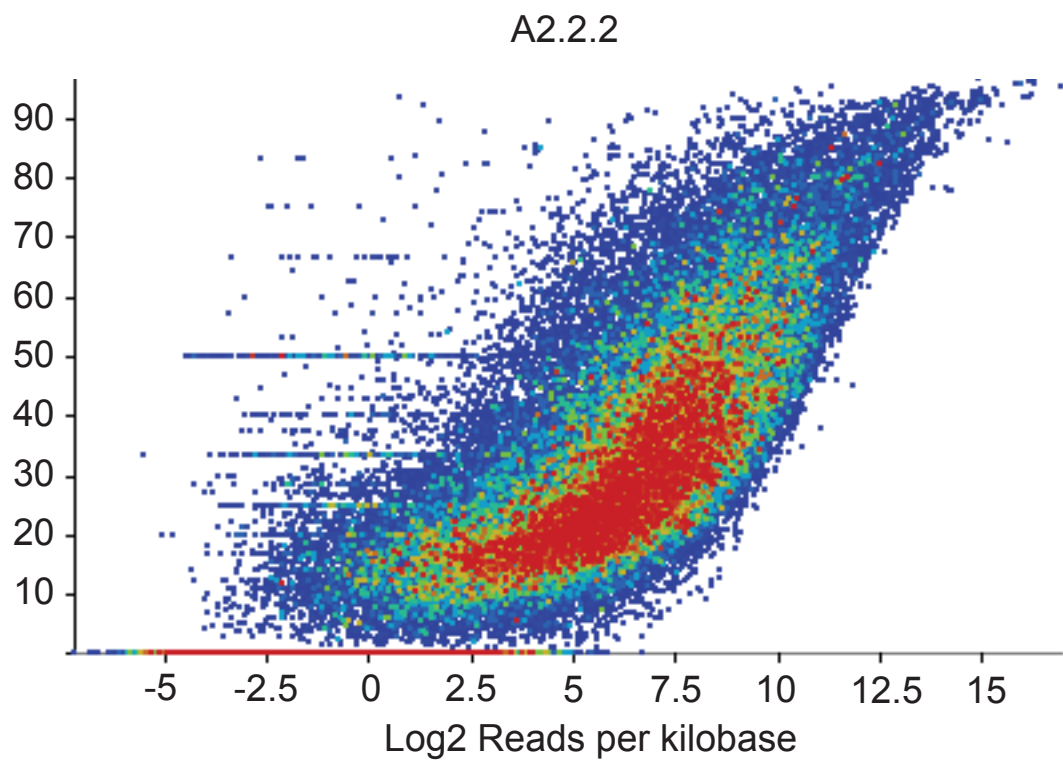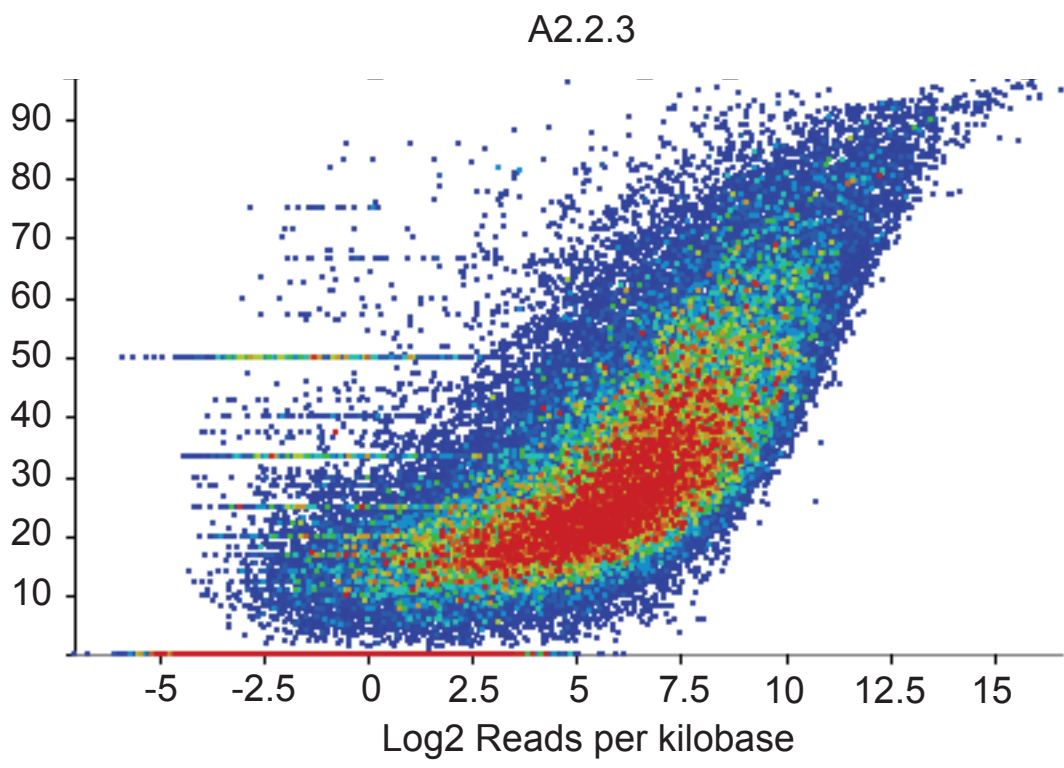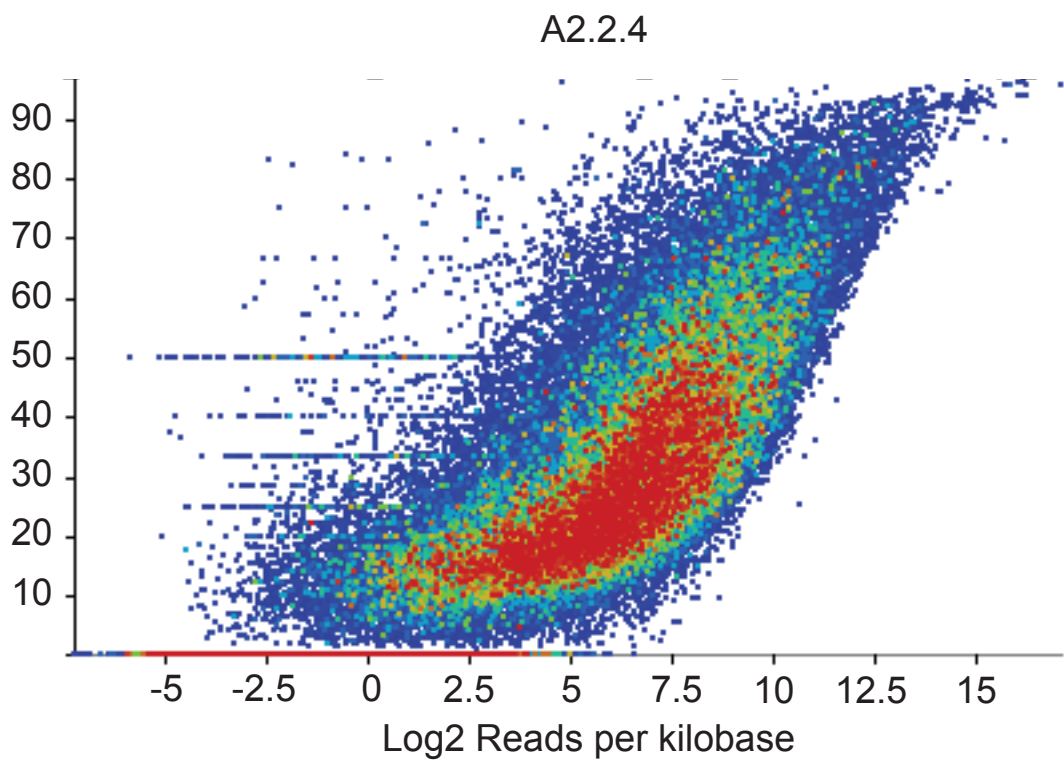

Spared B/D L4

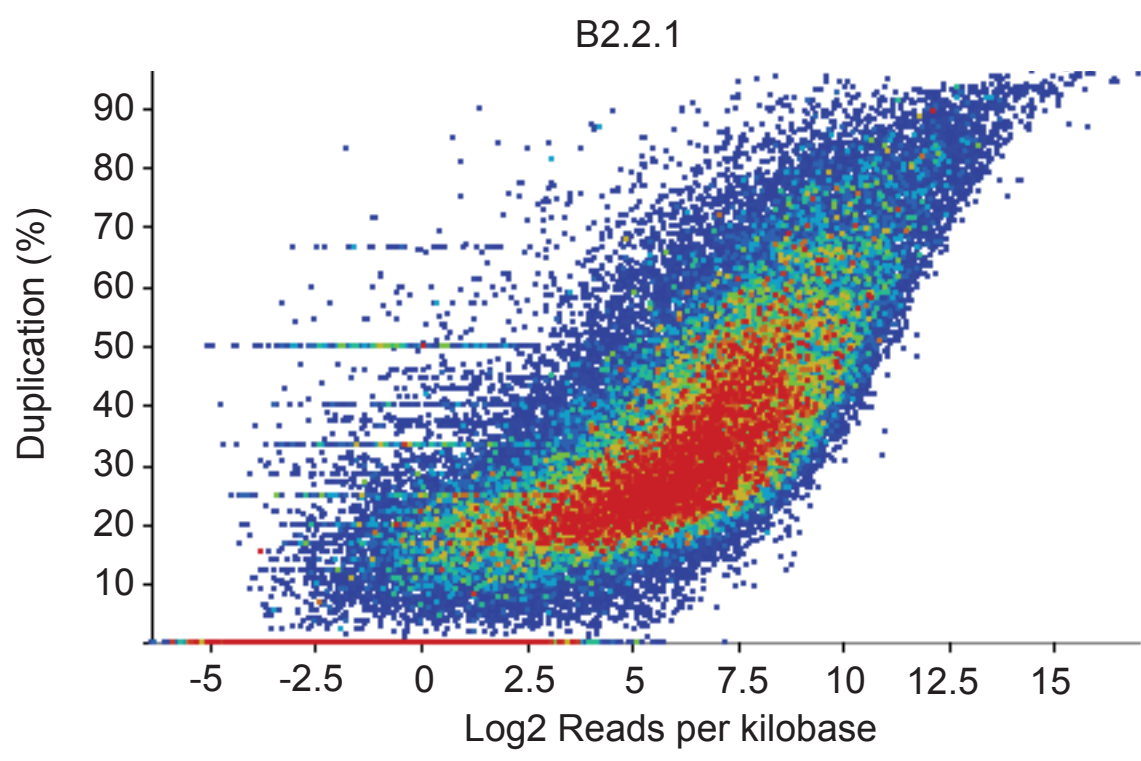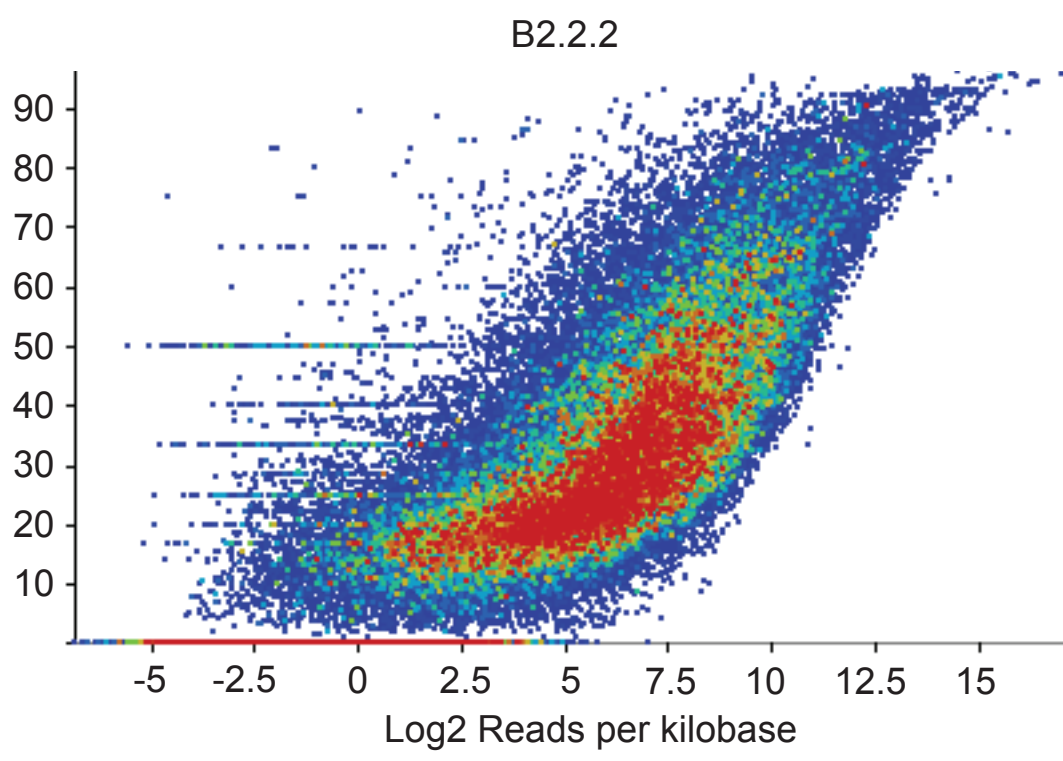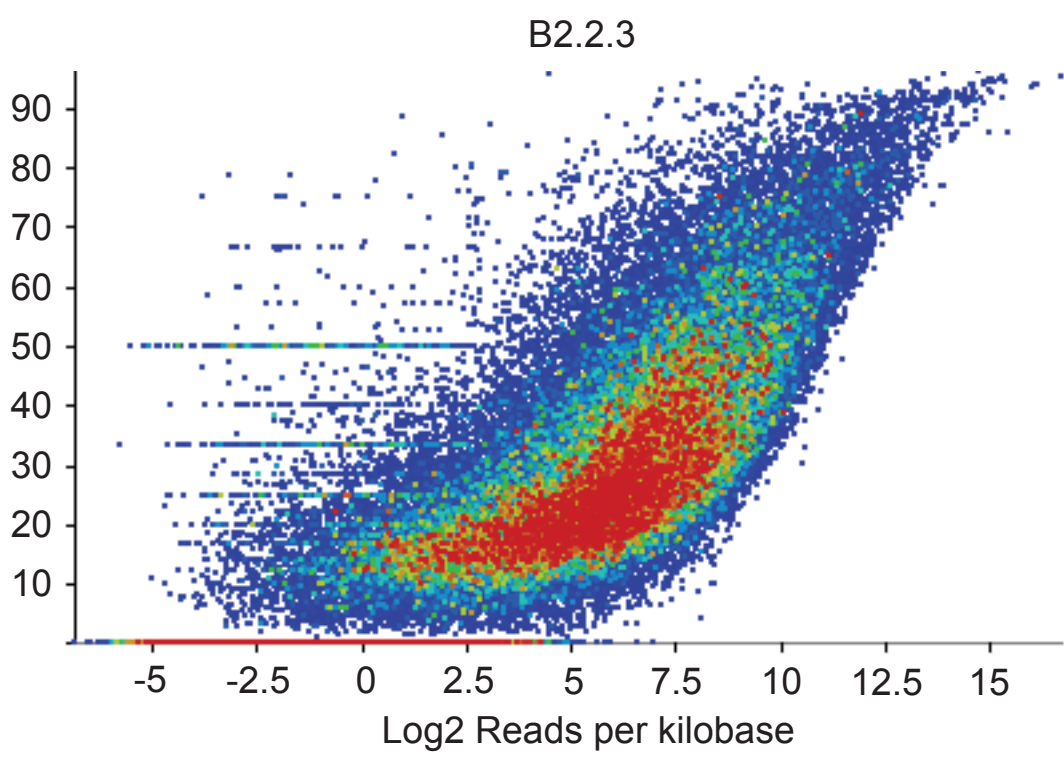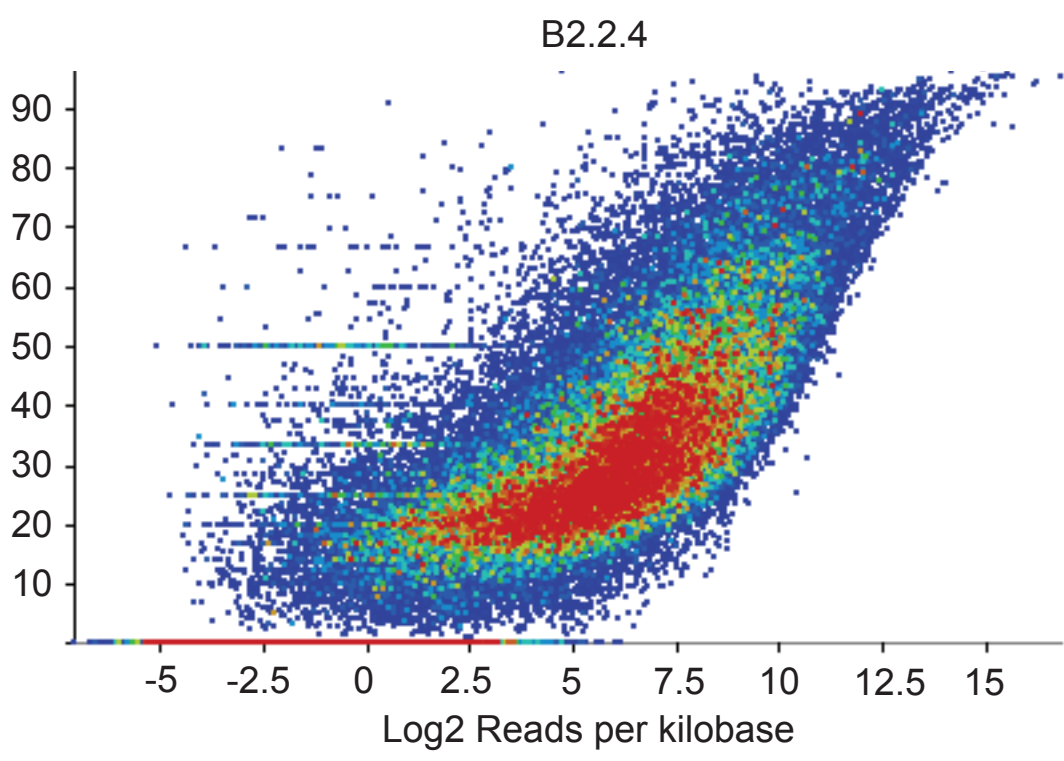

Spared A/E L2/3

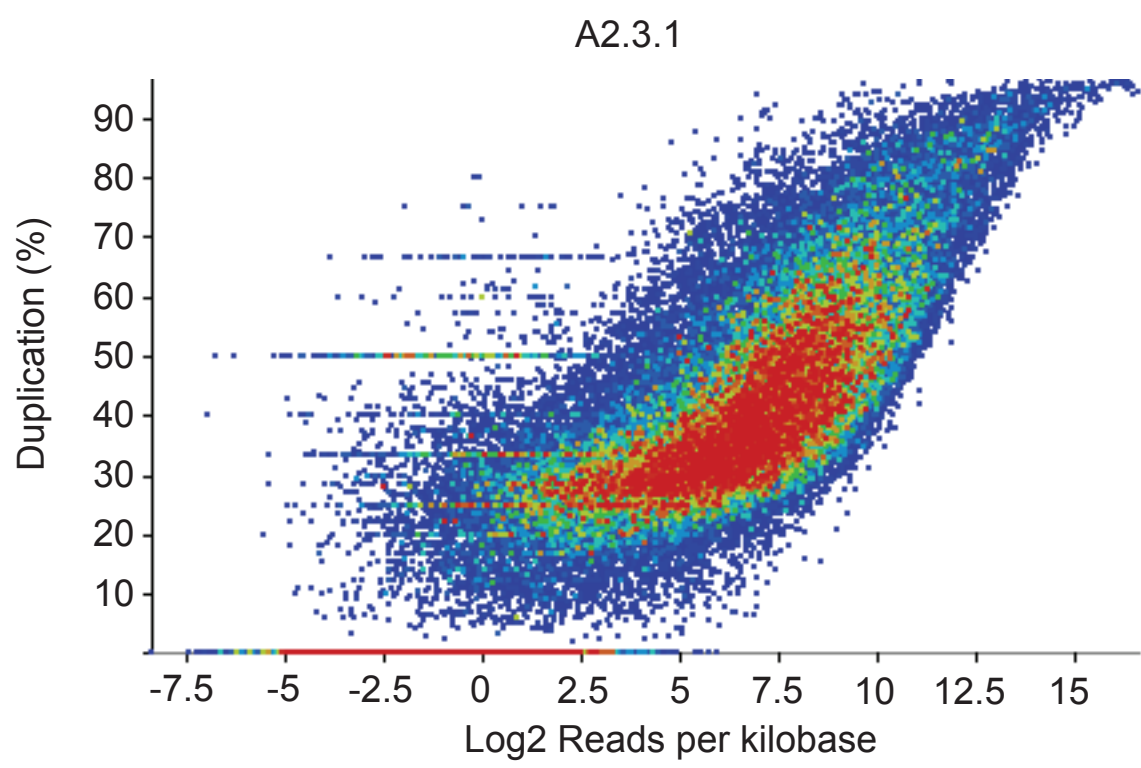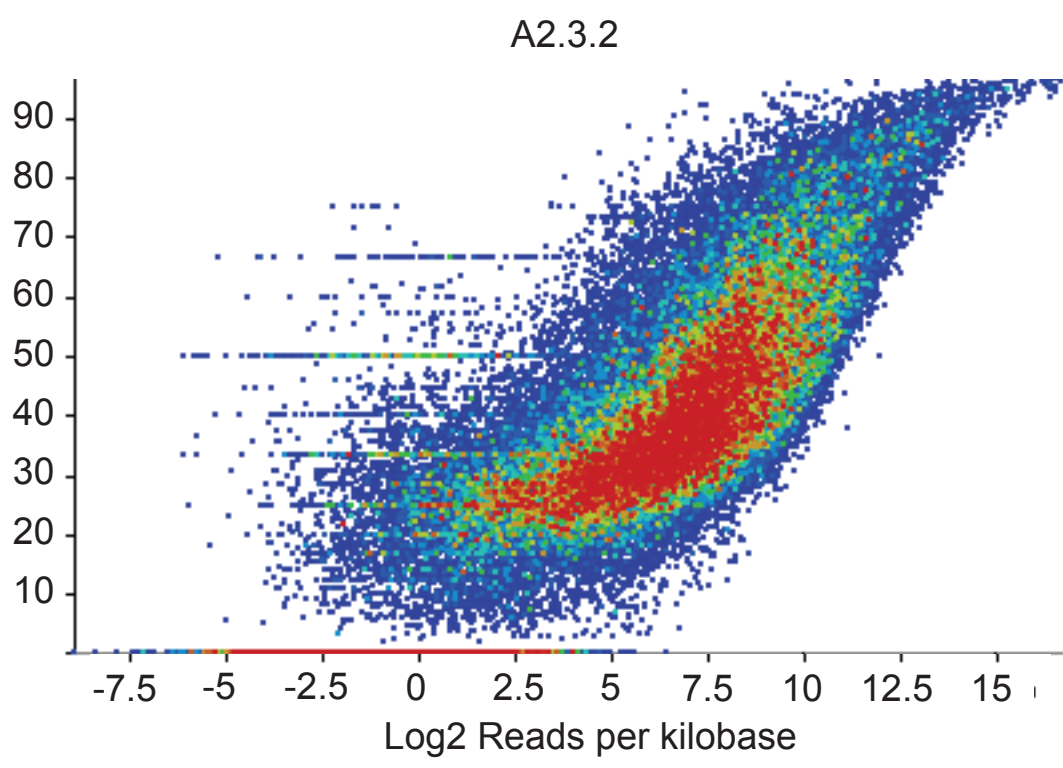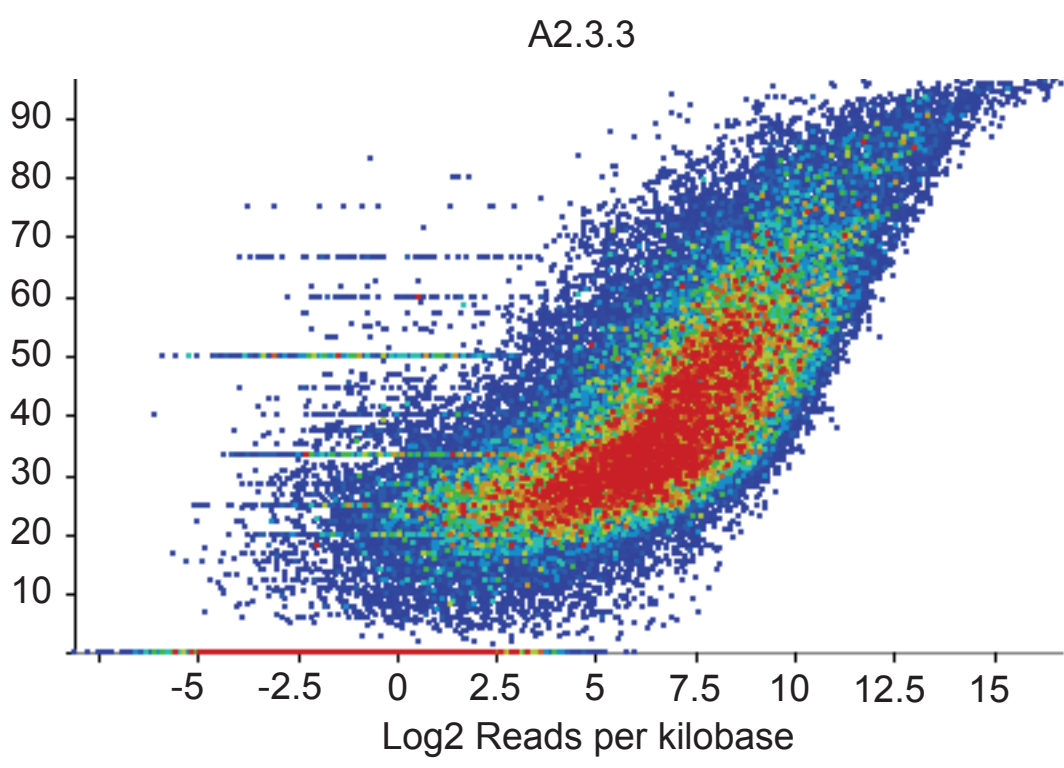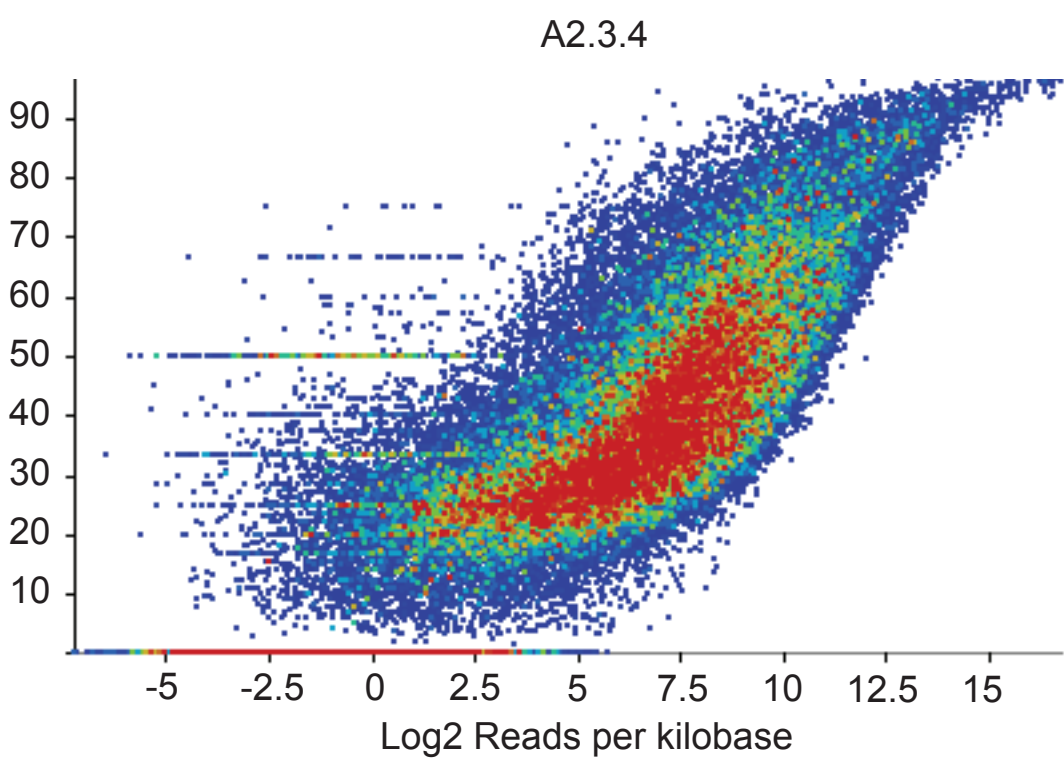

Spared A/E L4

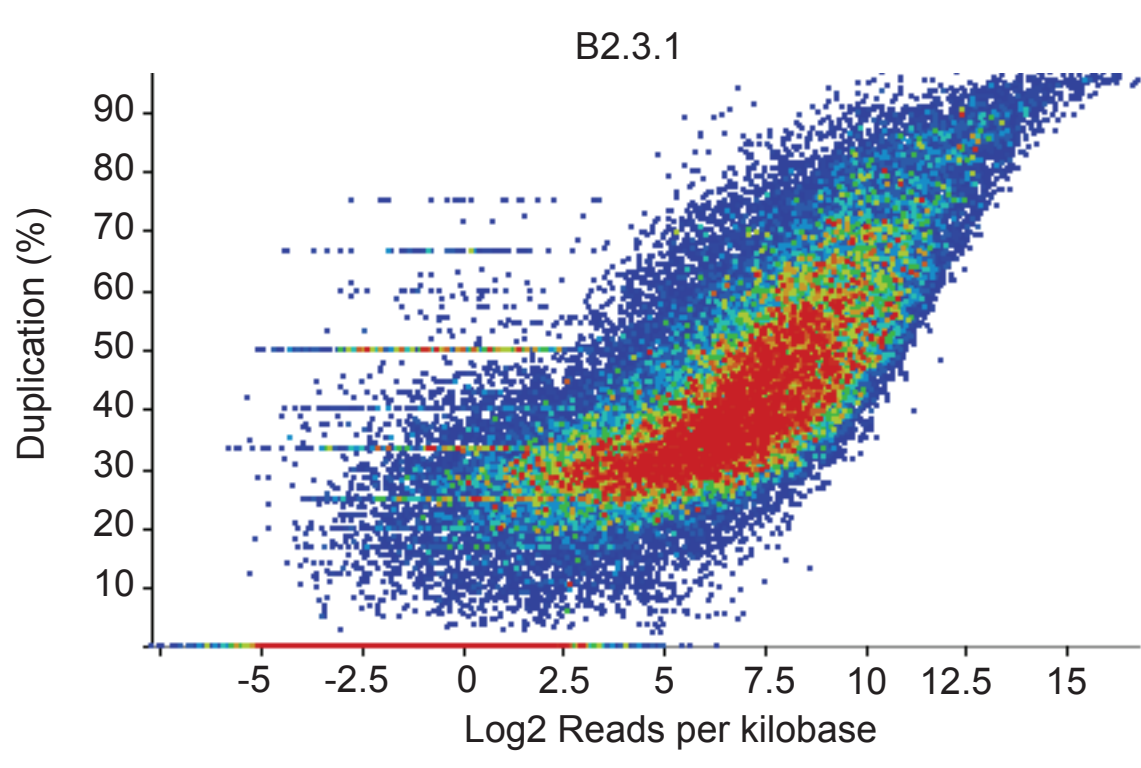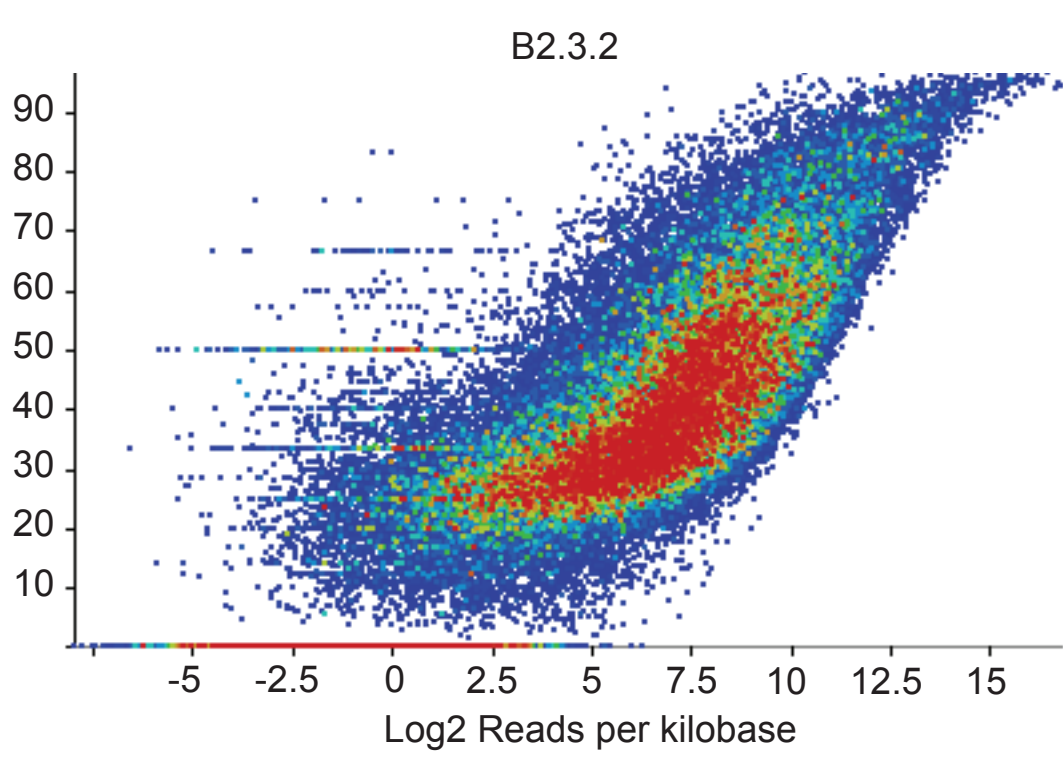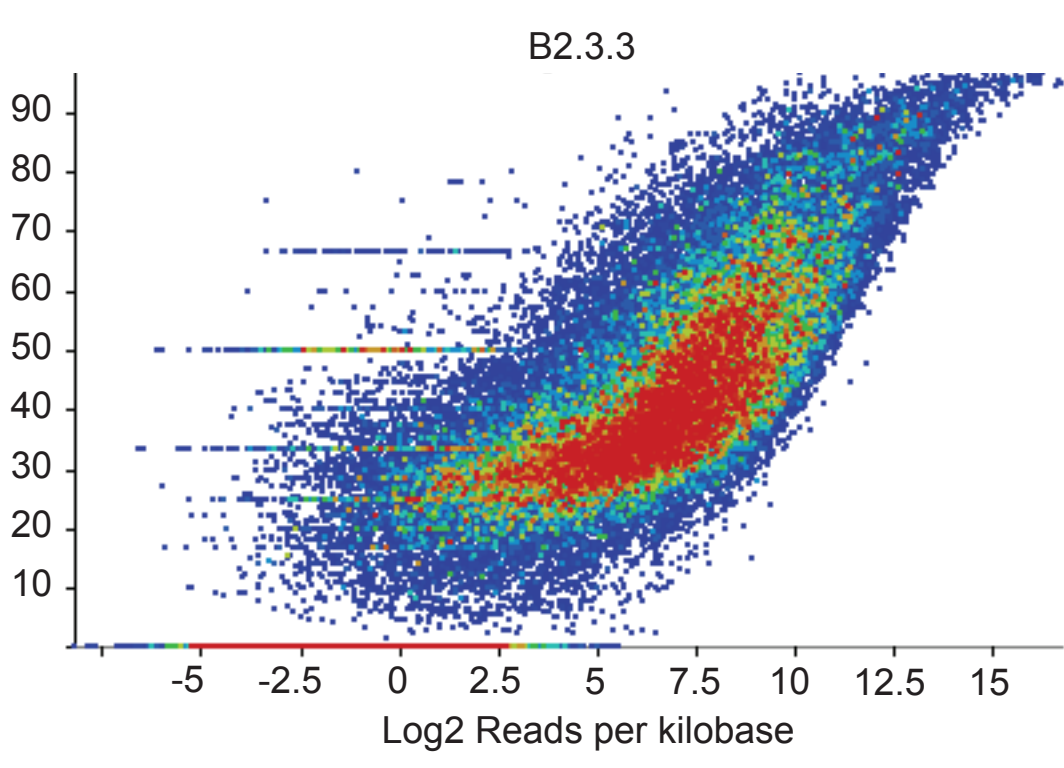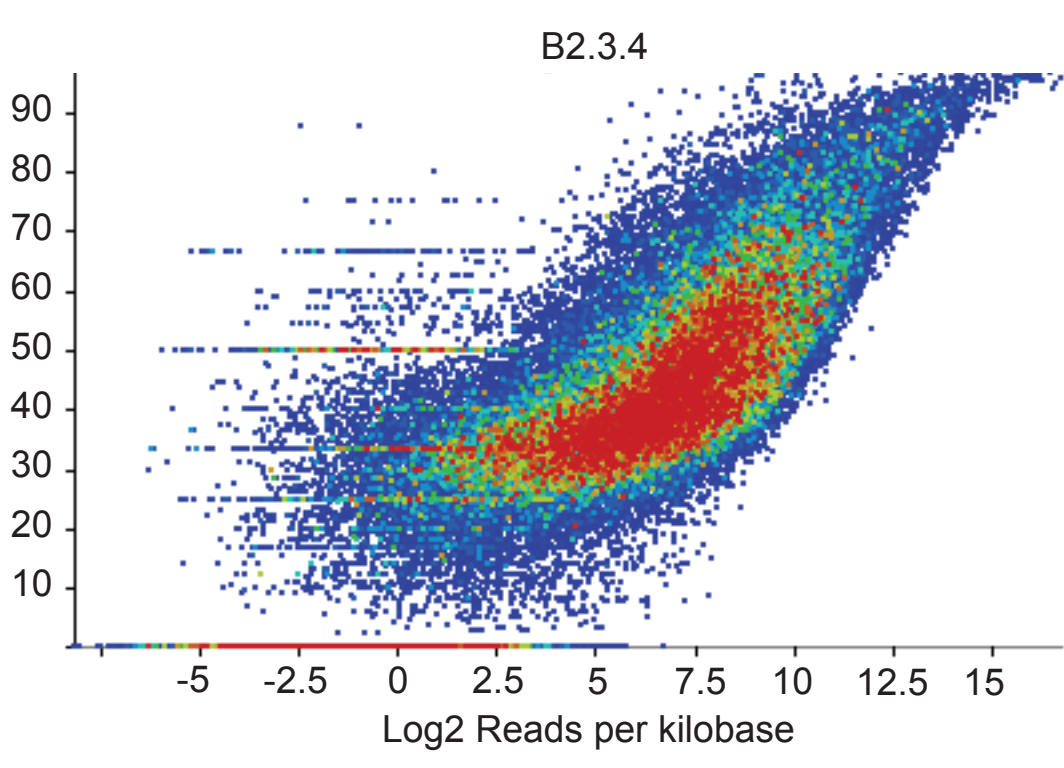

Supplement: Additional files [file gix081_supp.zip › SupplementalFigure1.pdf]

**A**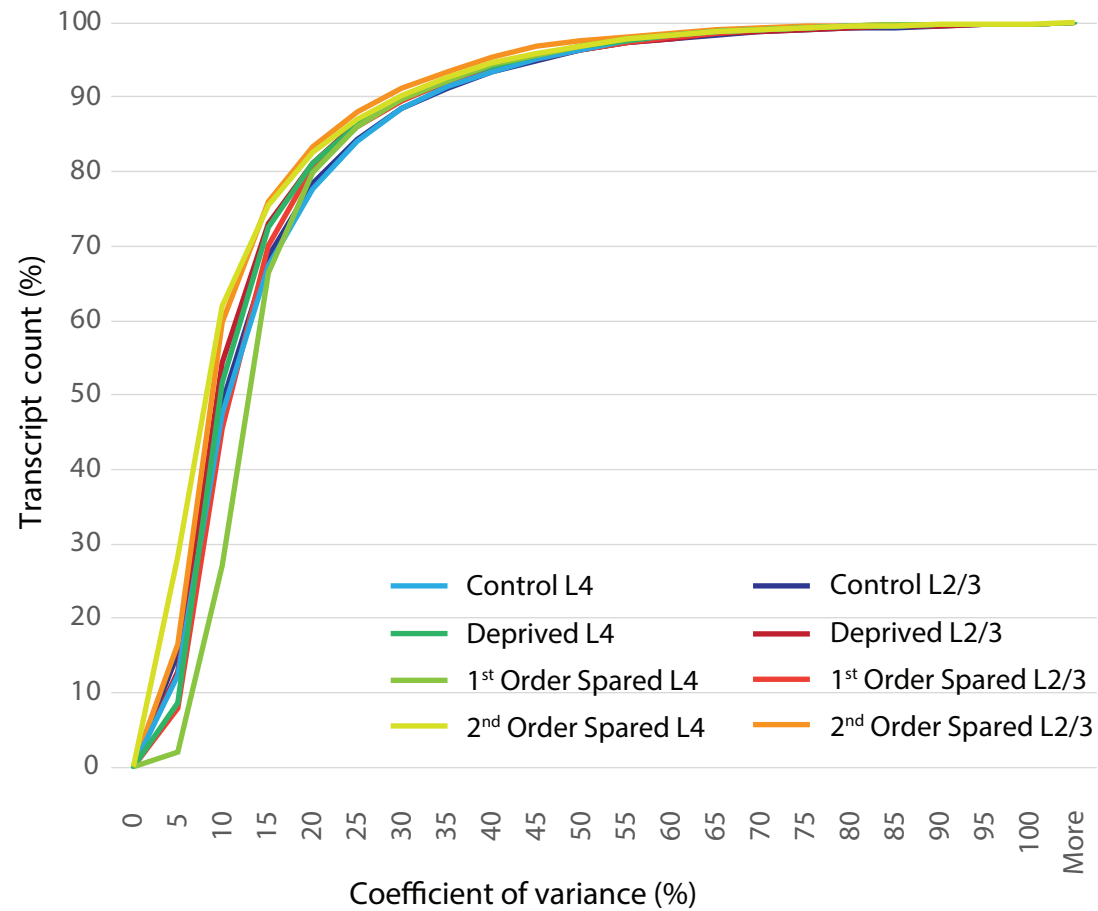**B**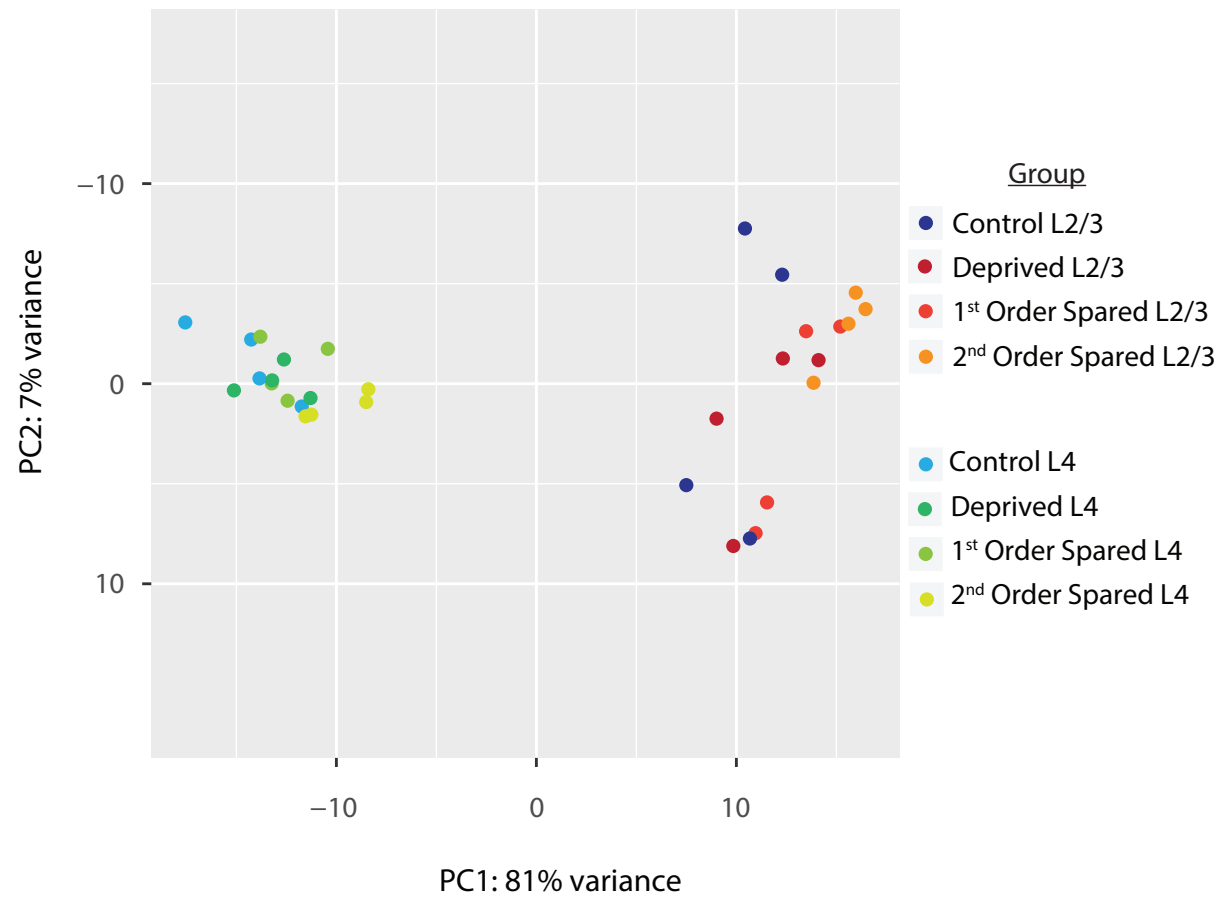

Supplement: Additional files [file gix081_supp.zip › SupplementalFigure2.pdf]
