# Supplementary material for: Transcriptional mapping of the primary somatosensory cortex upon sensory deprivation
Source: Gigascience. 2017 Aug 23;6(10):1–6. doi: 10.1093/gigascience/gix081 (PMC5965344; doi:10.1093/gigascience/gix081)
Supplement: Response-to-Reviewer-Comments_Revision-1.pdf [file gix081_Response-to-Reviewer-Comments_Revision-1.pdf]

Author's Response To Reviewer Comments

Dear Editor,

We would like to thank the reviewers for reading the revised manuscript. As requested, in the current version we have now added the RRIDs wherever possible.

Sincerely,

On behalf of the authors
Tansu Celikel
