# Supplementary material for: Transcriptional mapping of the primary somatosensory cortex upon sensory deprivation
Source: Gigascience. 2017 Aug 23;6(10):1–6. doi: 10.1093/gigascience/gix081 (PMC5965344; doi:10.1093/gigascience/gix081)
Supplement: Response-to-Reviewer-Comments_Revision-2.pdf [file gix081_Response-to-Reviewer-Comments_Revision-2.pdf]

Author's Response To Reviewer Comments

As requested, the comments by Dr. Nogoy have been addressed. Specifically, keywords and funding information were added, and the references have been checked.
