# Supplementary material for: Transcriptional mapping of the primary somatosensory cortex upon sensory deprivation
Source: Gigascience. 2017 Aug 23;6(10):1–6. doi: 10.1093/gigascience/gix081 (PMC5965344; doi:10.1093/gigascience/gix081)
Supplement: Reviewer-1-Report-(Original-Submission).pdf [file gix081_Reviewer-1-Report-(Original-Submission).pdf]

**Reviewer Report**

**Title:** Transcriptional mapping of the primary somatosensory cortex upon sensory deprivation

**Version:** Original Submission **Date:** 1/30/2017

**Reviewer name:** Mingshan Xue

**Reviewer Comments to Author:**

Kole and colleagues reported a transcriptome profiling dataset of the rodent barrel cortex upon sensory deprivation. This dataset is important and will be a helpful resource for studying experience-dependent plasticity, as the molecular mechanisms underlying this plasticity remains largely elusive. The methods for generating this dataset is appropriate and data quality is well controlled.Specific Point 1: According to Fig1, it seems that the control data are from the layer 2/3 and layer 4 of the C-row, but this is not explicitly stated in the text. Ideally, the cortical column (the row) that is deprived or spared should be compared to the same column from the controls. The authors should explain or justify why it is appropriate to compare the control C-row to the spared A/B/D/E-rows. Are there major transcriptional differences between columns?

**Level of Interest**

Please indicate how interesting you found the manuscript: An article of importance in its field

**Quality of Written English**

Please indicate the quality of language in the manuscript: Acceptable

**Declaration of Competing Interests**

Please complete a declaration of competing interests, considering the following questions:

- Have you in the past five years received reimbursements, fees, funding, or salary from an organisation that may in any way gain or lose financially from the publication of this manuscript, either now or in the future?
- Do you hold any stocks or shares in an organisation that may in any way gain or lose financially from the publication of this manuscript, either now or in the future?
- Do you hold or are you currently applying for any patents relating to the content of the manuscript?
- Have you received reimbursements, fees, funding, or salary from an organization that holds or has applied for patents relating to the content of the manuscript?
- Do you have any other financial competing interests?
- Do you have any non-financial competing interests in relation to this paper?

If you can answer no to all of the above, write 'I declare that I have no competing interests' below. If your reply is yes to any, please give details below.

I declare that I have no competing interests.

I agree to the open peer review policy of the journal. I understand that my name will be included on my report to the authors and, if the manuscript is accepted for publication, my named report including any attachments I upload will be posted on the website along with the authors' responses. I agree for my report to be made available under an Open Access Creative Commons CC-BY license (http://creativecommons.org/licenses/by/4.0/). I understand that any comments which I do not wish to be included in my named report can be included as confidential comments to the editors, which will not be published.

I agree to the open peer review policy of the journal
