# Supplementary material for: Transcriptional mapping of the primary somatosensory cortex upon sensory deprivation
Source: Gigascience. 2017 Aug 23;6(10):1–6. doi: 10.1093/gigascience/gix081 (PMC5965344; doi:10.1093/gigascience/gix081)
Supplement: Reviewer-2-Report-(Original-Submission).pdf [file gix081_Reviewer-2-Report-(Original-Submission).pdf]

Reviewer Report

Title: Transcriptional mapping of the primary somatosensory cortex upon sensory deprivation

Version: Original Submission **Date:** 2/14/2017

Reviewer name: Ben Huang

Reviewer Comments to Author:

Summary:
In this manuscript, the authors present a dataset of transcriptional profiles in mouse barrel cortex layer-2/3 and layer-4 neurons under control and sensory-deprived conditions (i.e. single whisker removal during postnatal development P12-24). The authors extracted RNAs from manually isolated layer-2/3 and layer-4 sections of cortical slices obtained from control and sensory-deprived mice, performed bulk-tissue RNA-seq, ran quality control, mapped the reads to the common reference database, and partially confirmed the promise of the method for identifying layer-specific transcripts by showing expressions of known layer-specific molecular markers. The premise for the study - using differential transcriptional profiling to identify molecular components underlying experience-dependent plasticity - is interesting and important. The quality of the RNA sequencing is nicely validated. However, it is unclear whether the data presented would be sufficiently powered to detect systematic differences in gene expression between the sensory-deprived and control conditions. It would be nice if the authors could clarify or address the following issues:

1. The number of replicates is not clearly stated. The n number of the experiments is not specified within the text; it is only labeled on Figure 1 (n=4 each for sensory-deprived and control conditions). Is this n of 4 from 4 mice, or from 4 slices of the same mouse? If it is from the same mouse, then it would be underpowered for detecting any systematic differences in expression, given the cross-animal variability.

2. To further validate the utility of the dataset, it would be nice to include graphs for visualizing the sources of variation in the data, such as principal component analysis (PCA). The clustering of samples by experimental conditions in the PCA plots would be a good indicator of the quality and usefulness of the dataset for answering questions regarding experience-dependent plasticity.

3. One important source of variation is the wide variety of cell types that are not distinguishable in the bulk tissue samples that were processed and sequenced. Expression changes in different types of excitatory principal neurons, inhibitory interneurons, and glial cells would all be included in the dataset, while the premise set out by the manuscript implies that the changes seen in the transcriptional profiles could be attributed to intracortical or translaminar excitatory projection neurons. It would be nice to clarify this limitation to the readers in the manuscript.

Level of Interest

Please indicate how interesting you found the manuscript: An article whose findings are important to those with closely related research interests

Quality of Written English

Please indicate the quality of language in the manuscript: Acceptable

Declaration of Competing Interests

Please complete a declaration of competing interests, considering the following questions:

- Have you in the past five years received reimbursements, fees, funding, or salary from an organisation that may in any way gain or lose financially from the publication of this manuscript, either now or in the future?
- Do you hold any stocks or shares in an organisation that may in any way gain or lose financially from the publication of this manuscript, either now or in the future?
- Do you hold or are you currently applying for any patents relating to the content of the manuscript?
- Have you received reimbursements, fees, funding, or salary from an organization that holds or has applied for patents relating to the content of the manuscript?
- Do you have any other financial competing interests?
- Do you have any non-financial competing interests in relation to this paper?

If you can answer no to all of the above, write 'I declare that I have no competing interests' below. If your reply is yes to any, please give details below.

I declare that I have no competing interests.

I agree to the open peer review policy of the journal. I understand that my name will be included on my report to the authors and, if the manuscript is accepted for publication, my named report including any attachments I upload will be posted on the website along with the authors' responses. I agree for my report to be made available under an Open Access Creative Commons CC-BY license (http://creativecommons.org/licenses/by/4.0/). I understand that any comments which I do not wish to be included in my named report can be included as confidential comments to the editors, which will not be published.

I agree to the open peer review policy of the journal
